# Novel One-Step Total Synthesis of *trans*-Dehydroosthol and Citrubuntin

**DOI:** 10.3390/molecules30051067

**Published:** 2025-02-26

**Authors:** Zhiwen Liu, Baoyue Ge, Xushun Gong, Fusheng Wang, Ting Lei, Shizhi Jiang

**Affiliations:** 1 College of Pharmacy, Dali University, Dali 671000, China; ldliuzhiwen@163.com (Z.L.); gby17662435129@163.com (B.G.); gongxushun@163.com (X.G.); wfsyn@163.com (F.W.); 2 Yunnan Provincial Key Laboratory of Entomological Biopharmaceutical R&D, Dali University, Dali 671000, China

**Keywords:** protecting-group-free, redox-neutral, one-pot (domino) Heck/dehydration reaction, isoprene, meroterpenoid

## Abstract

Efficient and simple syntheses of *trans*-dehydroosthol and citrubuntin were achieved in a single step by implementing a protecting-group-free, redox-neutral strategy that utilized readily available starting materials. In this approach, a practical one-pot (domino) Heck/dehydration reaction was carried out utilizing less reactive bromocoumarin, resulting in excellent stereoselectivity and atomic economy. Through the implementation of this new, efficient, and scalable synthesis method, the formal synthesis of a series of novel meroterpenoid natural products was successfully achieved.

## 1. Introduction

Meroterpenoids are hybrid natural products and a fascinating class of compounds that possess diverse chemical structures and intriguing biological activities [1], including antimicrobial [2], antiviral [3], anticancer [4], anti-inflammatory [5], and antioxidant properties [6]. They are derived from the fusion of terpenoid and non-terpenoid moieties, which provide them with distinct properties and potential applications in various fields, including pharmaceutical, medicinal, and agricultural industries [7]. Meroterpenoids have a wide range of sources [8], including plants, marine organisms, and microorganisms. Their unique structures enable them to interact with specific targets in living systems, making them valuable candidates for drug discovery and development. The significant biological activities and unique chemical structures of meroterpenoids have attracted great interest from synthetic and biosynthetic chemists. However, current synthesis strategies are not straightforward owing to the complexes processes involved and strongly depend on the not easily available key intermediate and expensive starting materials to produce the target molecule.

**Figure 1:**
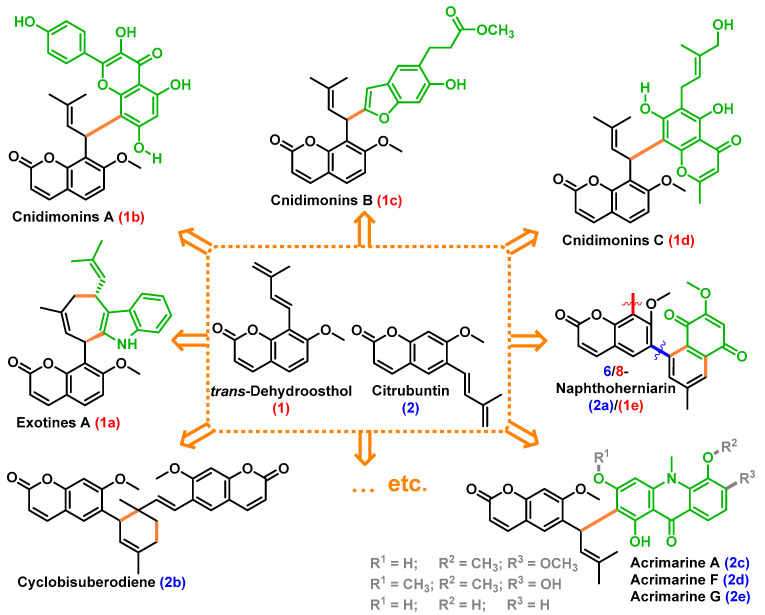
Structures of *trans*-dehydroosthol, citrubuntin, and several related meroterpenoids.

*Trans*-dehydroosthol (**1**) and its structurally related compound citrubuntin (**2**) are a class of coumarin derivatives with the same unique isoprene side chain. These active natural products can be found in many commonly used medicinal plants and act as crucial intermediates in the synthesis of diverse meroterpenoids (Figure 1). *Trans*-dehydroosthol was originally isolated from the leaves of *Murraya exotica L.* in 1987 [9] and later isolated from *Citrus* plants in 1993 [10] and *Murraya paniculata* in 2019 [11]. The impact of *trans*-dehydroosthol on NO production in BV-2 microglial cells was investigated. The findings indicated that *trans*-dehydroosthol possesses noteworthy inhibitory properties, suggesting its potential anti-inflammatory and analgesic effects [11]. First reported in 1980, “citrubuntin” was formed by the dehydration of suberenol and named as “(*E*)-suberodiene” [12]. Even though “citrubuntin” had not yet been isolated from natural resources at that time, the existence of glenadiene and gleinene in nature indicated that this compound may occur naturally. Citrubuntin was isolated from *Citrus grandis* for the first time by Wu [13] in 1988 and then from *Micromelum falcatum* by Wang [14] in 2019. *Micromelum falcatum* is a traditional herbal remedy in China, Thailand, and Vietnam. Its leaves and roots are utilized in these regions for various medicinal purposes, such as treating infected wounds, relieving pain, addressing rheumatism and muscular atrophy, reducing fever, and alleviating discomfort caused by insect bites [15]. Notably, coumarin components, which have been extensively studied, are the main bioactive compounds responsible for these therapeutic properties [16]. This finding suggests that citrubuntin may also exhibit good efficacy.

The chemical versatility of *trans*-dehydroosthol and citrubuntin enables the synthesis of various complex molecules with diverse biological activities and therapeutic potential. For example, synthetic chemists can modify and incorporate various dienophile (or conjugated diolefine), functional groups, and stereochemistry by strategically utilizing these two compounds as building blocks, streamlining synthesis paths toward numerous complex natural products. The total synthesis of these two isoprene-containing molecules has attracted the attention of numerous research groups [17,18,19,20,21], highlighting their significance in the field of meroterpenoid synthesis. Moreover, their synthesis aids in the quest for novel natural products with wide-ranging applications and contributes to the development of new drugs, flavors, and agricultural agents.

Given their significant pharmacological potential and unique structural features, trans-dehydroosthol and citrubuntin have emerged as compelling targets for synthetic exploration. These compounds belong to a broader class of biologically active meroterpenoids, which have attracted considerable attention in natural product synthesis. Recent studies have highlighted the structural diversity within this family, exemplified by notable examples such as exotines A (**1a**, indole-coumarin hybrid) [22], the cnidimonin series (**1b**–**d**, featuring flavonol-, benzofuran-, and chromone-coumarin frameworks) [23], 6/8-naphthoherniarin (**2a**/**1e**, quinone-coumarin conjugate) [24], cyclobisuberodiene (**2b**, coumarin dimer) [25], and the acrimarine group (**2c**–**e**, acridinone-coumarin hybrids) [26]. The development of efficient synthetic routes to these architecturally complex molecules remains crucial for advancing their biological evaluation and medicinal applications.

In 1988, Reisch and Murray [17,18] separately reported the initial total synthesis of *trans*-dehydroosthol, though the overall yields were not satisfactory. Their approach employed 8-iodo-7-methoxycoumarin as a crucial starting material. The highly reactive iodide was used as a coupling partner to introduce the 3-hydroxy-3-methyl-1-butenyl side chain, which served as a precursor for the isoprene unit. However, this method necessitated relatively high temperatures and extended reaction times (120 °C for 3 days; 90 °C for 24 hours under Jeffery conditions), even when using highly reactive iodides. Tedious dehydration reactions were performed using hazardous POCl_3_ or *p*-TsCl as the acylation agent and hot pyridine (reflux) or DIPEA as the reaction medium to obtain *trans*-dehydroosthol, with overall yields of 23.8% and 29%, respectively (calculated from the corresponding iodo-coumarin). In 2018, Martin [19,20] achieved the synthesis of *trans*-dehydroosthol in a relatively high overall yield by integrating the advantages of Reisch and Murray’s synthesis routes. Although this is a venerable transformation to the isoprene group in terms of yield (78%, two steps from iodo-coumarin; or 20%, four steps from 7-hydroxy coumarin), the use of highly active iodide cannot be avoided. According to the literature, the synthesis of 8-iodo-7-methoxycoumarin presents significant challenges. In the reported studies, inefficient processes such as iodination and subsequent methylation of the crude product were employed [27]. These methods resulted in very low conversions, necessitating the use of 7-hydroxycoumarin, which has a high structural similarity to the target compound. However, even with this approach, the desired iodide was obtained in yields of only 20%–30%, accompanied by a substantial amount of residual 7-methoxycoumarin. Furthermore, the instability of the iodide complicates its application, making the overall synthesis process highly complex.

Before being isolated and identified from the natural source, citrubuntin has been produced as an artificial molecule via POCl_3_ and the pyridine-promoted dehydration of suberenol and named as (*E*)-suberodiene. In 1990, Reisch [21] published a report on the first total synthesis of citrubuntin, using challenging brominated substrate as the coupling substrate. The key Heck coupling reaction was conducted under Jeffery conditions [28], resulting in the formation of the coupling product in 62% yield. Following the above protocol, citrubuntin was successfully synthesized under strong heating conditions using hazardous *p*-TsCl as the acylating reagent and troublesome pyridine as the solvent. Although moderate yields of coupling product can be obtained under Jeffery conditions (Pd(OAc)_2_, NaHCO_3_, *n*Bu_4_NBr), which is considered “a major achievement” for the Heck reaction [29], the need for a phase-transfer catalyst for stoichiometry is essential and troublesome because *n*Bu_4_NBr is associated with inconvenience work-up and purification processes. Wastewater containing a non-negligible amount of a phase-transfer catalyst produced in the reactions also restricts the application of these reactions on an industrial scale. Efforts to develop efficient, scalable, and environmentally friendly synthesis strategies for these two compounds are imperative to advance research in meroterpenoid synthesis and broaden the applications of related molecules.

## 2. Results

**Scheme 1:**
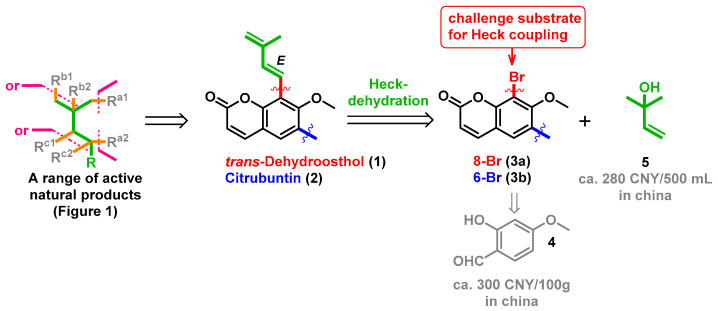
Retrosynthetic analyses of trans-dehydroosthol, citrubuntin, and a series of natural products.

Driven by the need for an efficient synthetic approach to produce trans-dehydroosthol and citrubuntin, which are crucial for the synthesis of numerous meroterpenoids, we sought an alternative strategy. Given the simplicity and efficiency of the one-pot (domino) Heck/dehydration reaction, we designed a novel retrosynthetic route as depicted in Scheme 1. We hypothesized that developing an effective catalyst system for the transition metal-catalyzed coupling of less reactive but cost-effective bromocoumarin would represent a significant improvement. This manuscript details our progress in achieving this goal.

Although the isoprene side chain is a relatively uncommon biogenetic modification of prenyl residues, it frequently appears in the *trans*-configuration in many valuable natural and chemical compounds. Our interest in natural products with an isoprene group dates to our synthesis of several useful natural products [30,31,32,33]. A crucial step in this synthesis involves a streamlined one-pot (domino) Heck/dehydration reaction, where the corresponding bromine reacts with 1,1-dimethyl allyl alcohol in the presence of a slightly low loading of base (1.0 equiv. trialkylamine).

**Scheme 2:**
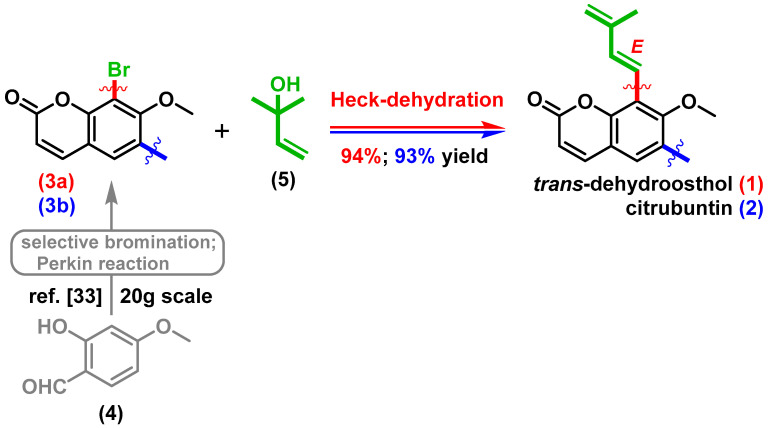
One-step syntheses of *trans*-dehydroosthol and citrubuntin.

In our retrosynthesis analyses of *trans*-dehydroosthol (**1**) and citrubuntin (**2**), we identified the need for a coupling reaction with 8/6-bromo coumarin (**3a/3b**) and 1,1-dimethyl allyl alcohol (**5**), as shown in Scheme 2. This strategy appeared highly feasible owing to the easy availability of starting materials to synthesize these two natural products in a single step. Our synthesis commenced with brominated coumarin as the key material, which is commercially available and can also be prepared readily on a dekagram scale using inexpensive 4-methoxysalicylaldehyde **4** via the regioselective bromination and CsOAc-promoted Perkin reaction optimized by our group [33].

### 2.1. Effect of the Bases

After obtaining multigram quantities of the known compound **3a**, we focused on the crucial reaction for the introduction of isoprene moiety. Initially, we used the conditions roughly based on the previously reported synthesis of murraol—8-bromocoumarin, 1,1-dimethylallyl alcohol, Pd(OAc)_2_, P(*o*-tol)_3_, Et_3_N (1.0 equiv.), toluene, and BHT (0.1 equiv.)—expecting that this low base and polymerization-inhibitor-containing version would facilitate the key C−C formation and the following cascade dehydration reaction. However, to our disappointment, this “standard” condition is completely ineffective, with only a trace of the desired product **1** observed after 3 h (Table 1, entry 1).

Even when the reaction temperature was raised to 120°C, the coupling reaction still only achieved a 35% yield of the desired product **1** after 10 hours (entry 2). This indicates that the one-pot (domino) Heck/dehydration process involving this brominated substrate is highly challenging. The high temperature required for the dehydration of the 1,1-dimethylallyl alcohol intermediate is likely due to the relatively unreactive nature of the hydroxyl group at the allylic position. This issue is further complicated by the strong electron-donating effects of the oxygen atoms in the two phenoxy groups (located in the *ortho*-position) on the conjugated system. Consequently, the rate-limiting step for one-pot (domino) Heck/dehydration, which is the dehydration of the 1,1-dimethylallyl alcohol moiety to the diene group, is slowed down, leading to the deceleration of the entire cascade reaction. When the reaction time was inadvertently prolonged to 31 h and the amount of base was deliberately reduced to 0.9 equiv., the coupling reaction produced the desired diene **1** in 67% and 68% yields (entry 1–2 and entry 3), respectively. Considering that the synthesis process starts from a less reactive substrate and involves a sequence of successive reactions, these yields from one-pot (domino) Heck/dehydration may be considerably good.

Basing on the preceding analysis and considering that the nature and amount of the base tremendously influence the Heck coupling reaction and subsequent tandem dehydration reaction, we assumed that the Heck alkenylation of our brominated substrate required the addition of a suitable base that not only mediates the electronic properties and the steric hindrance of the Pd center, but also alters the dehydration conditions (catalyzed by corresponding R_3_N·HBr). Therefore, we tested several readily available bases and discovered that upon replacing the trialkylamine from Et_3_N with (*n*-C_3_H_7_)_3_N and (*n*-C_8_H_17_)_3_N, the cascade reaction produced the desired product in 77% and 84% yields (entry 4–1 and entry 5–1), respectively. This phenomenon could be attributed to the more suitable electrical properties and steric hindrance nature of long alkyl-chain tertiary amines compared with triethylamine, possibly enhancing the reactivity of the oxidative insertion of the metal center into the deactivated Ar–Br bond. In addition, the increased steric demand of the catalyst complex also facilitates HBr dissociation in the reductive elimination step. The cascade dehydration reaction might also be enhanced. Conversely, other bases including DIPEA, (Cy)_2_NMe, DABCO, and DMAN exhibited lower efficiencies (entries 6–9). Bases such as *N*,*N*-diethylaniline and 2,6-lutidine did not facilitate the one-pot (domino) Heck/dehydration reaction (entries 10–11).

### 2.2. Effect of the Solvents

Inspired by our recent comprehensive investigation on the one-pot (domino) Heck/dehydration reaction and its streamlined procedure, we screened different solvents in combination with Pd(*t*-Bu_3_P)_2_ and Et_3_N to enhance the yield of this transformation. Among the solvents tested, a useful chloroalkane, DCE, yielded the best result (Table 2, entry 4). When the homolog of DCE, 1,3-DCP, was used as the reaction medium, a good yield was obtained after a short reaction time (90%, 10 h, entry 6–1). This optimization of solvent selection proved beneficial in improving the overall efficiency of the transformation. The use of other solvents, including xylene or chlorinated aromatics, chlorobenzene, and 2,4-DCT (entries 1–3), as well as CH_3_CN, DMF, DMAC, NMP, DMSO, THF, 2-MeTHF, 1,4-dioxane, DME, DEE, and *n*-BuOAc (entries 7–17), resulted in a moderate to significant decrease in yield. When DMSO was used as the reaction medium, yields of diene and allyl alcohol were unsatisfactory, presumably attributed to the significant polymerization or decomposition of the isopentenyl moiety during the coupling reaction. These observations highlight the importance of solvent selection in achieving optimal reaction efficiency and the good performance of DCE and its homolog 1,3-DCP as a solvent in this process.

Following the preliminary optimization of the one-pot (domino) Heck/dehydration reaction conditions (Table 1 and Table 2), we decided to combine the optimal bases with the best solvents to realize effective synthesis. According to the above experimental results (Table 2), the yield of diene **1** is relatively high when DCE and 1,3-DCP are used as the reaction media. Therefore, DCE and 1,3-DCP were selected as solvents and combined with (*n*-C_3_H_7_)_3_N or (*n*-C_8_H_17_)_3_N to improve the yield. Under the combination of DCE and (*n*-C_3_H_7_)_3_N, the yield of diene **1** reached up to 91% (or 93% yield on a Gram scale, Table 3, entry 2). However, the use of DCE (b.p. = 83.5 °C) as a solvent requires pressure-resistant equipment at a selected reaction temperature. In addition, the high boiling point of 1,3-DCP (b.p. = 120 °C–122 °C) facilitates various conventional heating reactions under normal atmospheric pressure. To further test the scalability of this methodology, we performed the reaction on a 1.2 g scale (4.7 mmol, Table 2, entries 6–2) with the suitable solvent–1,3-DCP. *Trans*-dehydroosthol was isolated in an excellent yield (94%) under atmospheric pressure, and the efficiency was greater than that obtained in the small-scale reaction (90%, Table 2, entries 6–1).

### 2.3. Further Application

Based on the aforementioned optimization of the one-pot (domino) Heck/dehydration reaction conditions, we synthesized analogs of trans-dehydroosthol and citrubuntin to evaluate their pharmacological activities and demonstrate the practicality of this synthetic approach. As illustrated in Table 4, the reaction proceeded very smoothly at 90 °C, yielding the desired diene **2** in excellent yield. The relatively low reaction temperature and time may be attributed to the reduced steric hindrance from the ortho-methoxy group (OMe) at the C-6 position of **3b**. While citrubuntin can be synthesized from (*E*)-suberenol via POCl_3_ and pyridine-promoted dehydration, a method previously reported through Jeffery-modified Heck coupling from **3b** [12], this route is less efficient than the strategy employed by our group. Additionally, the reaction was highly efficient on a gram scale under atmospheric pressure, achieving a yield of 97% (Table 4, entries 1–2). The application of this methodology to a broad scope of useful natural products is currently underway in our laboratory and will be reported in the future.

With the efficient synthesis of *trans*-dehydroosthol and citrubuntin in hand and to further demonstrate the usefulness of the proposed synthesis method, we attempted to synthesize several valuable meroterpenoid natural products **1a**, **1b**, **1c**, **1d**, **1e**, **2a**, **2b**, **2c**, **2d**, and **2e** (Figure 1). These desired products could be prepared in a single step from commercially available or readily available substrates and *trans*-dehydroosthol (or citrubuntin), according to the literature [22,23,24,25,26]. Our laboratory is currently undertaking endeavors to enhance the synthesis efficiency of the abovementioned hybrid natural products, and we anticipate that the results of these endeavors will be reported in the future.

## 3. Materials and Methods

### 3.1. General Information

NMR spectra were acquired using a Bruker AVANCE III instrument (400 MHz for ¹H and 100 MHz for ¹³C) in the specified solvent. Chemical shifts (*δ*) are reported in parts per million (ppm) relative to tetramethylsilane, with the solvent resonance serving as an internal reference. Deuterochloroform was used as the solvent in all cases. Coupling constants (*J*) are provided in Hertz (Hz). Multiplicity is described using the following abbreviations: s (singlet), d (doublet), t (triplet), q (quartet), dd (doublet of doublets), dt (doublet of triplets), AB q (AB-quartet), and m (multiplet). For ¹³C-NMR, the number of contributing carbon nuclei is indicated in parentheses following the chemical shift value. Reaction progress was monitored by thin-layer chromatography on 0.2-0.25 mm silica gel plates (GF-254) using UV light (254 nm) for visualization. Phosphomolybdic acid (10 wt.% in EtOH) was used as the visualizing agent. Flash chromatography was conducted using silica gel (200-300 mesh).

Unless otherwise specified, all chemicals and solvents were used as received without further purification. All of the following solvents and chemicals were obtained from Innochem (Beijing, China): 2-Methyl-3-buten-2-ol (98%), Bis(tri-tert-butylphosphine)palladium(0) (98%), Phosphomolybdic acid hydrate (98%), Ethanol (99.7%), Triethylamine (99%), *N*,*N*-Dipropyl-1-propanamine (99%), Tri-n-octylamine (97%), *N*,*N*-Diisopropylethylamine (99%), *N*,*N*-Dicyclohexylmethylamine (98%), 1,8-Bis(dimethylamino)naphthalene (98%), 1,4-Diazabicyclo [2.2.2]octane (98%), 2,6-Lutidine(98%), *N*,*N*-Diethylaniline (99%), Butylated hydroxytoluene (99%). Acetonitrile (99.9%), *N*,*N*-Dimethylformamide (99.9%), and Dimethyl sulfoxide (99.8%) are extra dry solvents (with molecular sieves) and were used as received. In addition, Chlorobenzene (99%), Xylene (99%), Dichloroethane (98.9%), 1,2-Dichloropropane (99%), 1,3-Dichloropropane (99%), *N*,*N*-Dimethylacetamid (≥99%), N-Methylpyrrolidone (99.5%), Ethylene glycol diethyl ether (98%), Dichloromethane (≥99.5%), 1,2-Dimethoxyethane (99.5%), Tetrahydrofuran (99%), 1,4-Dioxane (99%), and 2-Methyltetrahydrofuran (99%) are all analytical reagents. An appropriate amount of calcium hydride was added and refluxed for 3 hours prior to use. Then, the fraction was taken out and degassed twice with a nitrogen-filled balloon. The resulting anhydrous and oxygen-free reagent was stored over molecular sieves under nitrogen. Petroleum ether and ethyl acetate were both industrial grade products that had been reevaporated using a rotary evaporator before use. The silica gel for the chromatography (0.2–0.25 mm, 50 × 100 mm, GF254) was obtained from Qingdao Haiyang Chemical Co., Ltd. (Qingdao, China).

### 3.2. Synthesis of 3-Bromo-2-hydroxy-4-methoxybenzaldehyde and 5-Bromo-2-hydroxy-4-methoxybenzaldehyde

#### 3.2.1. Synthesis of 3-Bromo-2-hydroxy-4-methoxybenzaldehyde

Under a nitrogen atmosphere, a round-bottom flask equipped with a magnetic stir bar was charged with a solution of aldehyde **4** (20 g, 131 mmol) in dichloromethane (410 mL). The solution was cooled to −78 °C, followed by the dropwise addition of TiCl_4_ (18 mL, 157 mmol, pure; or 157 mL of a 1 M solution in dichloromethane) over 20 min. Next, a solution of bromine (6.7 mL, 131 mmol) in dichloromethane (100 mL) was added dropwise over 30 min. The reaction mixture was stirred at −78 °C for 1 hour and then allowed to warm to room temperature over 2 hours. When TLC monitoring indicated a complete reaction (as monitored by TLC, about 5 h), a saturated aqueous solution of Na_2_SO_3_ (100 mL) was added to the resulting mixture and stirred for another 10 min. The mixture was separated, and the aqueous phase was extracted with CH_2_Cl_2_ (2 × 750 mL). The combined organic phases were washed with brine (100 mL), dried over Na_2_SO_4_, filtered, and concentrated in vacuo at 30 °C. After purification by flash column chromatography using petroleum ether/ethyl acetate (100:1 to 15:1) as the eluent, the title compound was obtained as a colorless solid (26.9 g, 89%), m.p. 115–117 °C. ^1^H NMR (400 MHz, CDCl_3_) *δ*_H_: 11.93 (s, 1H), 9.71 (s, 1H), 7.51 (d, *J* = 8.7 Hz, 1H), 6.62 (d, *J* = 8.7 Hz, 1H), 3.99 (s, 3H); ^13^C NMR (101 MHz, CDCl_3_) *δ*_C_: 194.43, 162.83, 160.20, 134.69, 116.19, 103.87, 99.69, 56.96; IR (KBr): 3731.00, 2991.06, 2873.84, 2348.90, 1637.36, 1479.51, 1361.26, 1200.23, 829.10, 763.08, 551.18 cm^−1^; HRMS (EI) calcd for C_8_H_7_BrO_3_ [M − H]^−^ 228.9506, found 228.9506.

#### 3.2.2. Synthesis of 5-Bromo-2-hydroxy-4-methoxybenzaldehyde

Under a nitrogen atmosphere, a round-bottom flask equipped with a magnetic stir bar was charged with a solution of aldehyde **4** (20 g, 131 mmol) in dichloromethane (410 mL). To this solution, bromine (6.7 mL, 131 mmol) dissolved in dichloromethane (100 mL) was added dropwise at room temperature over 30 minutes. The reaction mixture was stirred continuously, and progress was monitored by TLC until completion (approximately 3 hours). Upon completion, a saturated aqueous solution of Na_2_SO_3_ (100 mL) was added, and the mixture was stirred for an additional 10 minutes. The mixture was separated, and the aqueous phase was extracted with CH_2_Cl_2_ (2 × 750 mL). The organic phases were washed with brine (100 mL), dried over Na_2_SO_4_, filtered and concentrated in vacuo at 30 °C. After purification by flash column chromatography (petroleum ether (PE): ethyl acetate (EA) = 100:1 to 25:1), the title compound was obtained as a colorless solid (26.4 g, 87%), m.p. 126–128 °C. ^1^H NMR (400 MHz, CDCl_3_) *δ*_H_: 11.43 (s, 1H), 9.68 (s, 1H), 7.67 (s, 1H), 6.47 (s, 1H), 3.94 (s, 3H); ^13^C NMR (101 MHz, CDCl_3_) *δ*_C_: 193.80, 163.79, 162.66, 137.36, 115.85, 102.25, 100.50, 56.90; IR (KBr): 3423.16, 2949.95, 2849.27, 2347.53, 1635.93, 1499.15, 1361.11, 1289.78, 1240.24, 1062.87, 759.71, 645.90, 539.88 cm^−1^; HRMS (EI) calcd for C_8_H_7_BrO_3_ [M − H]^−^ 228.9504, found 228.9506.

#### 3.2.3. Synthesis of 8-Bromo-7-methoxy-2*H*-chromen-2-one-**3a**

Under a nitrogen atmosphere, a Heavy-Wall Pressure Vessel equipped with a magnetic stir bar was charged with 3-bromo-4-methoxysalicylaldehyde (20 g, 87 mmol), CsOAc (16.7 g, 87 mmol), and acetic anhydride (70 mL). The vessel was sealed and heated in an oil bath at 160 °C. The reaction progress was monitored by TLC and completed after approximately 12 h. The mixture was then cooled to room temperature, diluted with ethyl acetate (1000 mL), and washed sequentially with hot water (5 × 100 mL) and brine (120 mL). The organic layer was dried over sodium sulfate, filtered, and concentrated under reduced pressure at 40 °C to yield the crude product. After purification by flash column chromatography (PE:EA = 10:1 to 5:1), the title compound **3a** was obtained as a yellow powder solid (15.5 g, 70%), m.p. 165–167 °C. ^1^H NMR (400 MHz, CDCl_3_) *δ*_H_: 7.62 (d, *J* = 9.5 Hz, 1H), 7.41 (d, *J* = 8.7 Hz, 1H), 6.87 (d, *J* = 8.7 Hz, 1H), 6.28 (d, *J* = 9.5 Hz, 1H), 3.99 (s, 3H); ^13^C NMR (101 MHz, CDCl_3_) *δ*_C_: 160.31, 159.30, 152.43, 143.30, 127.68, 113.96, 113.91, 108.09, 99.87, 56.99; IR (KBr): 3426.02, 3061. 00, 2346.67, 1728.29, 1600.08, 1278.14, 1076.71, 925.20, 839.62, 632.05, 577.89 cm^−1^; HRMS (EI) calcd for C_10_H_7_O_3_Br [M + Na]^+^ 276.9497, found 276.9477.

#### 3.2.4. Synthesis of 6-Bromo-7-methoxy-2*H*-chromen-2-one-**3b**

Under a nitrogen atmosphere, a Heavy-Wall Pressure Vessel equipped with a magnetic stir bar was charged with 5-bromo-4-methoxysalicylaldehyde (20 g, 87 mmol), CsOAc (16.7 g, 87 mmol), and acetic anhydride (70 mL). The vessel was sealed and heated in an oil bath at 160 °C. The reaction progress was monitored by TLC and completed after approximately 12 hours. The mixture was then cooled to room temperature, diluted with ethyl acetate (1000 mL), and washed sequentially with hot water (5 × 100 mL) and brine (120 mL). The organic layer was dried over sodium sulfate, filtered, and concentrated under reduced pressure at 40 °C to yield the crude product. After purification by flash column chromatography (PE:EA = 35:1 to 10:1), the title compound **3b** was obtained as a yellow powder solid (16.2 g, 73%), m.p. 196–198 °C. ^1^H NMR (400 MHz, CDCl_3_) *δ*_H_: 7.66 (s, 1H), 7.59 (d, *J* = 9.5 Hz, 1H), 6.84 (s, 1H), 6.30 (d, *J* = 9.5 Hz, 1H), 3.97 (s, 3H); ^13^C NMR (101 MHz, CDCl_3_) *δ*_C_: 160.60, 158.68, 155.03, 142.41, 131.51, 114.30, 113.40, 107.70, 100.31, 56.90; IR (KBr): 3420.10, 3285.39, 3066.57, 2344.55, 1731.58, 1601.17, 1371.41, 1261.69, 1211.35, 1036.02, 890.98, 693.81, 513.00 cm^−1^; HRMS (EI) calcd for C_10_H_7_O_3_Br [M + H]^+^ 254.9687, found 254.9651.

### 3.3. General Procedure for Table 1, Table 2, Table 3 and Table 4

Under a nitrogen atmosphere, a double-neck round-bottom flask or a Heavy-Wall Pressure Vessel was equipped with a magnetic stir bar, a condenser, and charged with **3a** or **3b** (50 mg, 0.2 mmol, 1.0 eq.), Pd(*t*-Bu_3_P)_2_ (10 mg, 0.02 mmol, 0.1 eq.), BHT (4.3 mg, 0.02 mmol, 0.1 eq.), solvent (1.5 mL, degassed with nitrogen for 10 min prior to use), base (37 μL, 0.2 mmol, 1.0 eq.), and 1,1-dimethylallyl alcohol **5** (92 μL, 0.9 mmol, 4.5 eq.). The flask or vessel was then placed in an oil bath. Once the reaction was complete (monitored by TLC or at a predetermined time), the mixture was cooled to room temperature, and a saturated aqueous NaHCO_3_ solution (1 mL) was added and stirred for an additional 5 min. The mixture was filtered through a pad of celite and washed with ethyl acetate (EA, 30 mL). The filtrate was diluted with EA (50 mL), washed with water and brine, dried over Na_2_SO_4_, filtered, and concentrated in vacuo at 30 °C to yield the crude product. Purification by flash column chromatography (PE: EA gradient from 8:1 to 2:1) afforded the desired compound.

#### 3.3.1. Synthesis of *trans*-Dehydroosthol (**1**): Table 2, Entry 6–1 (Gram Scale)

Under a nitrogen atmosphere, a double-neck round-bottom flask or a Heavy-Wall Pressure Vessel was equipped with a magnetic stir bar, a condenser, and charged with **3a** (1.2 g, 4.7 mmol), Pd(*t*-Bu_3_P)_2_ (240 mg, 0.47 mmol), BHT (103.2 mg, 0.47 mmol), 1,3-Dichloropropane (36 mL, bubbled with nitrogen for 10 min prior to addition), Et_3_N (653 μL, 4.7 mmol), and 1,1-dimethylallyl alcohol **5** (2.2 mL, 21 mmol). The flask was then immersed in an oil bath preheated to 110 °C. The reaction progress was monitored by TLC, and after approximately 10 hours, the mixture was cooled to room temperature. A saturated aqueous NaHCO_3_ solution (10 mL) was added, and the mixture was stirred for 5 minutes. The resulting mixture was filtered through a celite pad and washed with ethyl acetate (100 mL). The filtrate was diluted with additional ethyl acetate (300 mL) and washed sequentially with water (240 mL) and brine (40 mL). The organic layer was dried over sodium sulfate, filtered, and concentrated under reduced pressure at 30 °C to yield the crude product. Purification by flash column chromatography (petroleum ether/ethyl acetate gradient, 8:1 to 2:1), the title compound *trans*-dehydroosthol was obtained as a yellow powder solid (1.07 g, 94% yield), m.p. 77–79 °C, and the compound **6** was obtained as a yellow powder solid (24.5 mg, 2%), m.p. 120–122 °C. Data for compound *trans*-dehydroosthol (**1**): ^1^H NMR (400 MHz, CDCl_3_) *δ*_H_: 7.62 (d, *J* = 9.5 Hz, 1H), 7.49 (d, *J* = 16.5 Hz, 1H), 7.30 (d, *J* = 8.6 Hz, 1H), 6.90 (d, *J* = 16.5 Hz, 1H), 6.86 (d, *J* = 8.6 Hz, 1H), 6.27 (d, *J* = 9.5 Hz, 1H), 5.19 (d, *J* = 2.2 Hz, 1H), 5.14 (t, *J* = 1.8 Hz, 1H), 3.97 (s, 3H), 2.03 (d, *J* = 1.3 Hz, 3H); ^13^C NMR (101 MHz, CDCl_3_) *δ*_C_: 161.12, 160.36, 152.67, 144.06, 143.20, 138.25, 127.08, 118.56, 117.28, 114.35, 113.22, 113.10, 107.68, 56.28, 18.47; IR (KBr): 3423.32, 3065.99, 2941.21, 2346.60, 1720.73, 1596.93, 1478.99, 1256.29, 1109.88, 1031.74, 840.74, 587.96, 474.00 cm^−1^; HRMS (EI) calcd for C_15_H_14_O_3_ [M + Na]^+^ 265.0837, found 265.0835.

Data for compound **6**: ^1^H NMR (400 MHz, CDCl_3_) *δ*_H_: 7.62 (d, 1H, *J* = 9.4 Hz), 7.30 (d, 1H, *J* = 8.6 Hz), 7.02 (d, 1H, *J* = 16.5 Hz), 6.93 (d, 1H, *J* = 16.5 Hz), 6.86(d, 1H, *J* = 8.6 Hz), 6.25(d, 1H, *J* = 9.4 Hz), 3.94 (s, 3H), 1.46(s, 6H); ^13^C NMR (101 MHz, CDCl_3_) *δ*_C_: 161.14, 160.32, 152.69, 144.59, 144.07, 127.16, 114.40, 113.72, 113.12, 113.05, 107.66, 71.77, 56.24, 30.00; IR (KBr): 3735.67, 3466.37, 2960.23, 2347.90, 1707.97, 1598.36, 1471.26, 1252.81, 1182.87, 1087.44, 823.12, 703.70, 562.82 cm^−1^; HRMS (EI) calcd for C_15_H_16_O_4_ [M + Na]^+^ 283.1038, found 283.1053.

#### 3.3.2. Synthesis of Citrubuntin (**2**): Table 4, Entry 1–2 (Gram Scale)

Under a nitrogen atmosphere, a double-neck round-bottom flask or a Heavy-Wall Pressure Vessel was equipped with a magnetic stir bar, a condenser, and charged with **3b** (1.2 g, 4.7 mmol), Pd(*t*-Bu_3_P)_2_ (240 mg, 0.47 mmol), BHT (103.2 mg, 0.47 mmol), 1,3-Dichloropropane (36 mL, bubbled with nitrogen for 10 min prior to addition), Et_3_N (653 μL, 4.7 mmol), and 1,1-dimethylallyl alcohol **5** (2.2 mL, 21 mmol). The flask was then immersed in an oil bath preheated to 90 °C. The reaction progress was monitored by TLC, and after approximately 8 hours, the mixture was cooled to room temperature. A saturated aqueous NaHCO_3_ solution (10 mL) was added, and the mixture was stirred for 5 minutes. The resulting mixture was filtered through a celite pad and washed with ethyl acetate (100 mL). The filtrate was diluted with additional ethyl acetate (300 mL) and washed sequentially with water (240 mL) and brine (40 mL). The organic layer was dried over sodium sulfate, filtered, and concentrated under reduced pressure at 30 °C to yield the crude product. Purification by flash column chromatography (petroleum ether/ethyl acetate gradient, 15:1 to 5:1), the title compound citrubuntin was obtained as a yellow powder solid (1.07 g, 97% yield), m.p. 176–178 °C. ^1^H NMR (400 MHz, CDCl_3_) *δ*_H_: 7.65 (d, *J* = 9.5 Hz, 1H), 7.55 (s, 1H), 6.87 (d, *J* = 16.3 Hz, 1H), 6.79 (d, *J* = 16.3 Hz, 1H), 6.78 (s, 1H), 6.27 (d, *J* = 9.5 Hz, 1H), 5.13 (d, *J* = 2.1 Hz, 1H), 5.10 (t, *J* = 1.7 Hz, 1H), 3.92 (s, 3H), 1.99 (d, *J* = 1.2 Hz, 3H); ^13^C NMR (101 MHz, CDCl_3_) *δ*_C_: 161.26, 160.05, 155.21, 143.63, 142.40, 133.21, 124.93, 124.37, 121.74, 117.94, 113.54, 112.43, 99.10, 56.17, 18.73; IR (KBr): 3427.83, 3063.80, 2930.04, 2345.77, 1725.98, 1610.08, 1368.16, 1270.11, 1123.71, 1001.52, 865.87, 819.85, 458.49 cm^−1^; HRMS (EI) calcd for C_15_H_14_O_3_ [M + Na] ^+^ 265.0837, found 265.0835.

Data for compound **7**, m.p. 172–174 °C: ^1^H NMR (400 MHz, CDCl_3_) *δ*_H_: 7.63 (d, *J* = 9.5 Hz, 1H), 7.48 (s, 1H), 6.85 (d, *J* = 16.2 Hz, 1H), 6.77 (s, 1H), 6.36 (d, *J* = 16.2 Hz, 1H), 6.26 (d, *J* = 9.5 Hz, 1H), 3.90 (s, 3H), 1.44 (s, 6H); ^13^C NMR (101 MHz, CDCl_3_) *δ*_C_: 161.30, 160.03, 155.22, 143.60, 139.30, 125.44, 123.88, 119.84, 113.49, 112.31, 99.05, 71.36, 56.11, 30.02; IR (KBr): 3838.15, 3732.92, 3433.47, 2932.10, 2345.14, 1728.08, 1611.93, 1356.06, 1210.07, 1020.71, 830.13, 675.92 cm^−1^; HRMS (EI) calcd for C_15_H_16_O_4_ [M + Na]^+^ 283.0940, found 283.0941.

## 4. Conclusions

In summary, we developed a simple, practical method for preparing *trans*-dehydroosthol and citrubuntin in just one single step using the readily available starting materials. The naturally occurring compounds obtained in almost quantitative yield exhibited excellent stereoselectivity and atomic economy. This strategy offers several notable advantages: (1) the concise and efficient syntheses are characterized by a successful PGF (protecting group-free) approach and a redox-neutral strategy and (2) a highly active catalyst system has been developed for the one-pot (domino) Heck/dehydration reaction of deactivated coumarin substrates. This system is based on the electron-rich and bulky catalyst Pd[P(*t*-Bu)_3_]_2_, in combination with either Et_3_N or the less commonly used trialkylamine (*n*-C_3_H_7_)_3_N, and mediated by chlorinated solvents such as 1,3-DCP or DCE. Importantly, this synthetic approach not only enables the formal synthesis of novel dimeric natural products, including exotines A, cnidimonins A–C, 6/8-naphthoherniarin, cyclobi-suberodiene, and acrimarines A, F, and G, but also facilitates their overall synthesis. Ongoing efforts are focused on refining the practical, cost-effective conditions and methodologies for these dimeric compounds. The synthetic strategies reported in this study are being explored for potential application in related natural products. Further information on this development will be provided in the future.

## Figures and Tables

**Table 1:**
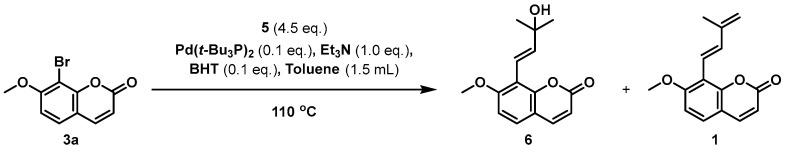
Study on the effect of the bases.

Entry	Variable	t[h]	3a (%) ^a^	6 (%) ^a^	1 (%) ^a^
1 ^b^	None	3 ^b^/31	-	97/32	2/67
2 ^b^	120 °C	10	-	63	35
3	Et_3_N (0.9) ^c^	18	-	31	68
4	(*n*-C_3_H_7_)_3_N (1.0/0.9) ^c^	21/29	-/-	31/22	69/77
5	(*n*-C_8_H_17_)_3_N (1.0/0.9) ^c^	29/22	-/-	22/4	75/84
6	DIPEA	9	-	25	67
7	(Cy)_2_NMe ^d^	27	-	89	10
8	DABCO	13	-	67	32
9	DMAN ^e^ (0.85) ^c^	31	4	39	56
10	N,N-Diethylaniline	5	64	-	28
11	2,6-Lutidine	10	71	-	27

“-” = Not detected. ^a^ Isolated yield. ^b^ Sealed tube. ^c^ The equiv. of the base. ^d^ *N*,*N*-Dicyclohexylmethylamine. ^e^ 1,8-Bis(dimethylamino)naphthalene.

**Table 2:**
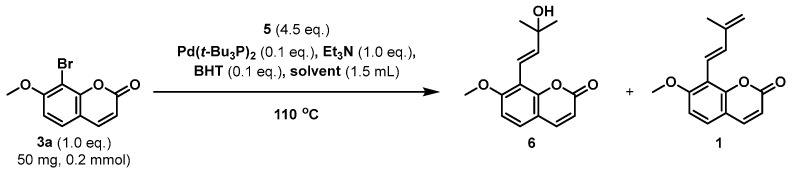
Study on the effect of the solvents.

Entry	Variable	t[h]	3a (%) ^a^	6 (%) ^a^	1 (%) ^a^
1	Xylene	27	-	78	20
2	Chlorobenzene	6	9	5	70
3	2,4-DCT ^b^	22	36	21	27
4 ^c^	DCE (1.0/0.9) ^d^	21/8	-/-	4/-	82/86
5 ^c^	1,2-DCP ^e^	30	-	31	61
6	1,3-DCP ^f^ (1.0/0.9) ^d^	10(10 ^g^)/14	-(-)/11	3(2)/16	90(94)/69
7 ^c^	CH_3_CN	21	-	61	36
8	DMF	17	-	47	48
9	DMAC	18	-	47	47
10	NMP	18	-	47	39
11	DMSO	19	-	12	26
12 ^c^	THF	21	-	33	66
13 ^c^	2-MeTHF ^h^	22	-	38.1	51.8
14 ^c^	1,4-Dioxane	15	-	15	77
15 ^c^	DME	22	-	60.7	35.3
16	DEE ^i^	20	-	58	30
17	*n*-BuOAc ^j^	21	-	74	25

^a^ Isolated yield. ^b^ 2,4-Dichlorotoluene. ^c^ Sealed tube. ^d^ The equiv. of the base. ^e^ 1,2-Dichloropropane. ^f^ 1,3-Dichloropropane. ^g^ Gram scale (1.2 g, 4.7 mmol). ^h^ 2-Methyltetrahydrofuran. ^i^ Ethylene glycol diethyl ether. ^j^ *n*-Butyl acetate.

**Table 3:**
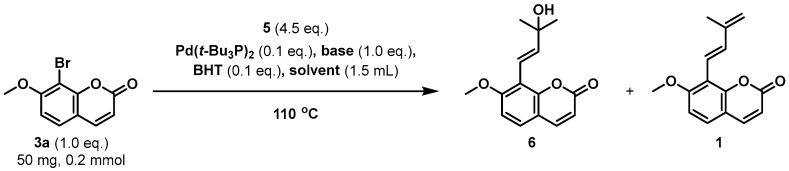
Study on the effect of the combination of solvents and bases.

Entry	Variable	t[h]	3a (%) ^a^	6 (%) ^a^	1 (%) ^a^
1 ^b^	DCE, (*n*-C_8_H_17_)_3_N (1.0/0.9) ^c^	18/7	-/-	10/2	84/89
2 ^b^	DCE, (*n*-C_3_H_7_)_3_N (1.0/0.9) ^c^	14(14 ^d^)/11	-(-)/-	4(-)/7	91(93)/83
3	1,3-DCP, (*n*-C_8_H_17_)_3_N (1.0/0.9) ^c^	21.5/21	-/-	35/28	64/70
4	1,3-DCP, (*n*-C_3_H_7_)_3_N (1.0/0.9) ^c^	26/16	-/-	15/23	78/67

^a^ Isolated yield. ^b^ Sealed tube. ^c^ The equiv. of the base. ^d^ Gram scale (1.2 g, 4.7 mmol).

**Table 4:**
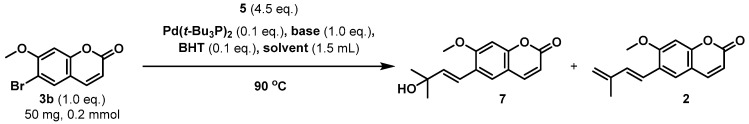
Study on the synthesis of citrubuntin (based on Table 2, entry 6-1 and Table 3, entry 2–1).

Entry	Variable	t[h]	3b (%) ^a^	7 (%) ^a^	2 (%) ^a^
1	1,3-DCP, Et_3_N	8/8 ^c^	-/-	4/-	94/97
2 ^b^	DCE, (*n*-C_3_H_7_)_3_N	8/8 ^c^	-/-	-/-	97/98

^a^ Isolated yield. ^b^ Sealed tube, 110 °C. ^c^ Gram scale (1.2 g, 4.7 mmol).

## Data Availability

The data underlying this study are available in the published article and its online Supplementary Materials.

## Supplementary Materials

The following supporting information can be downloaded at https://www.mdpi.com/article/10.3390/molecules30051067/s1, Figures S1–S16: ^1^H and ^13^C NMR spectra of the products; Figures S17–S24: ^1^H NMR Spectra for crude **6** and *trans*-dehydroosthol **(1)**; Tables S1–S4: ^1^H and ^13^C NMR spectral comparisons for compounds. References [9,17,21,27] are cited in the Supplementary Materials.
