# Supplementary material for: Novel One-Step Total Synthesis of trans-Dehydroosthol and Citrubuntin
Source: Molecules. 2025 Feb 26;30(5):1067. doi: 10.3390/molecules30051067 (PMC11901576; doi:10.3390/molecules30051067)
Supplement: Supplementary file 1 [file molecules-30-01067-s001.zip › molecules-3454766-supplementary.pdf]

molecules

MDPI

Supplementary Materials

Novel one-step total synthesis of *trans*-Dehydroosthol and Citrubuntin

Table of Contents:

^{1}H, ^{13}C NMR Spectra for All Products 3-bromo-2-hydroxy-4-methoxybenzaldehyde, 5-bromo-2-hydroxy-4-methoxybenzaldehyde, **3a**, **3b**, **6**, **7**, *trans*-dehydroosthol **(1)** and Citrubuntin **(2)**.. [2-9]

^{1}H NMR Spectra for crude **6** and *trans*-dehydroosthol **(1)** .... [10-13]

^{1}H and ^{13}C NMR spectral comparisons for compounds .... [14-17]

Reference .... [17]

NMR Spectra for All Compounds 3-bromo-2-hydroxy-4-methoxybenzaldehyde, 5-bromo-2-hydroxy-4-methoxybenzaldehyde, **3a**, **3b**, **6**, **7**, *trans*-dehydroosthol **(1)** and citrubuntin **(2)**

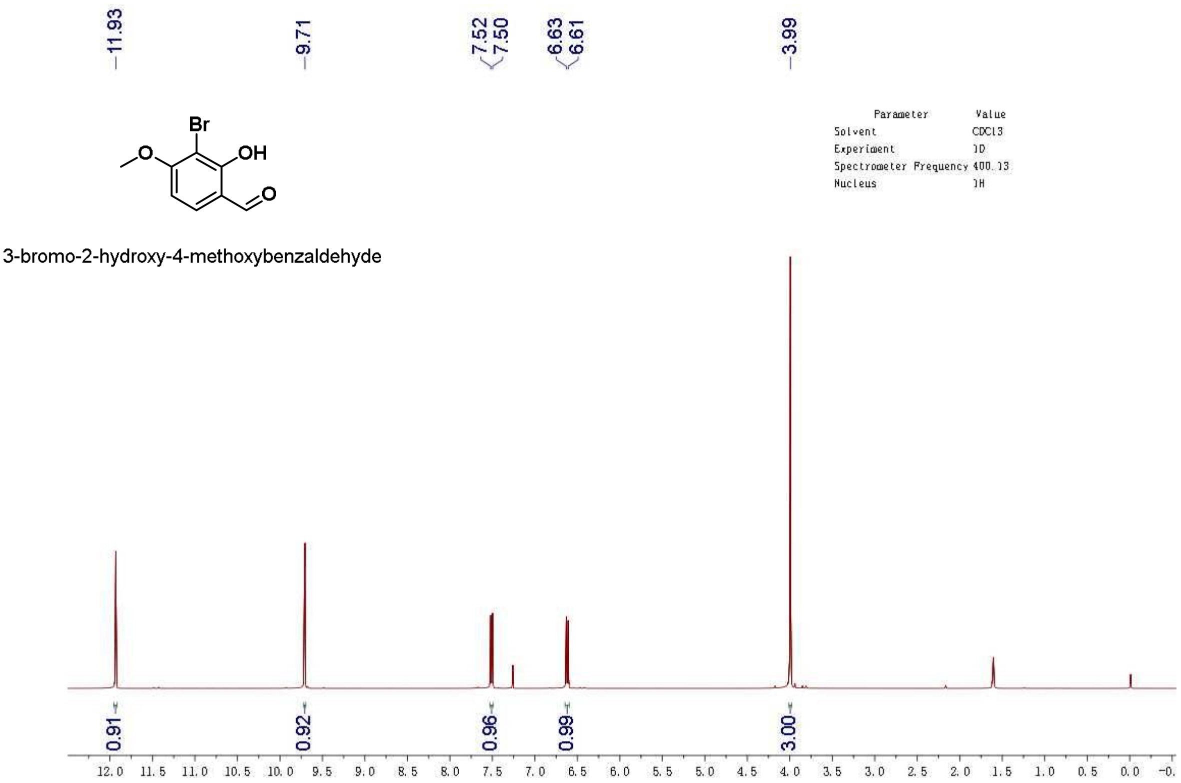

Figure S1. ^{1}H NMR spectrum of 3-bromo-2-hydroxy-4-methoxybenzaldehyde.

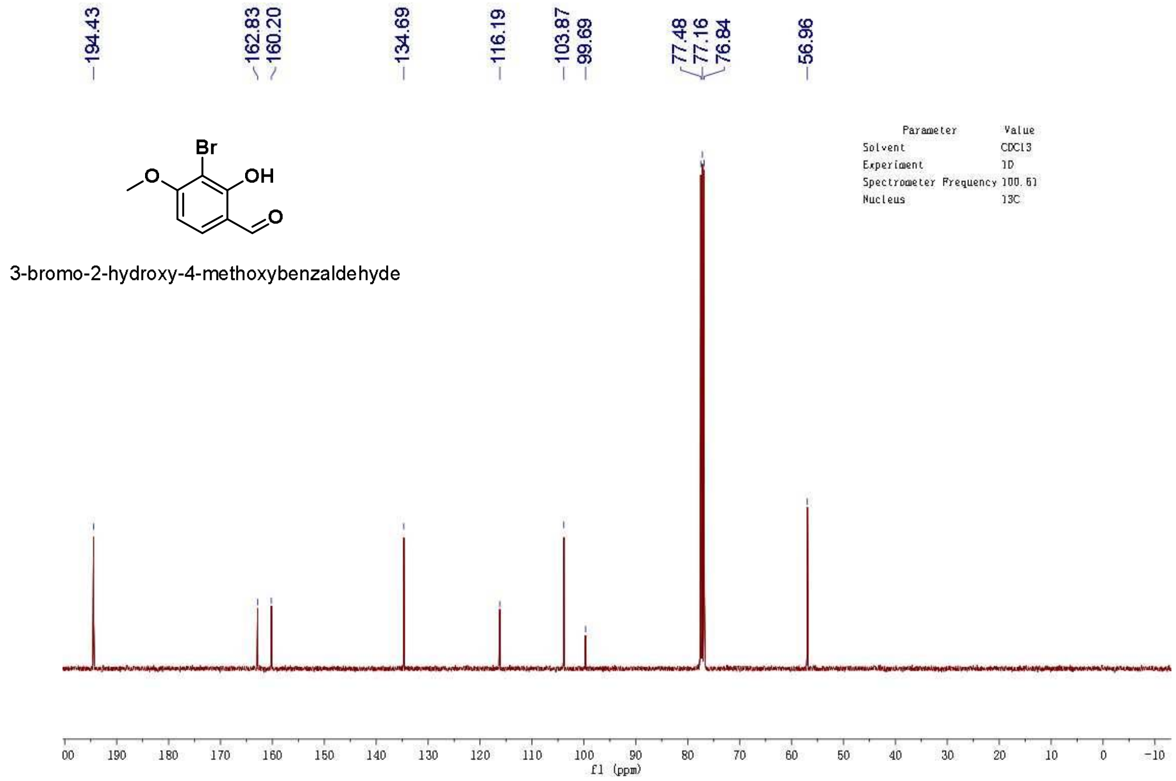

Figure S2. ^{13}C NMR spectrum of 3-bromo-2-hydroxy-4-methoxybenzaldehyde.

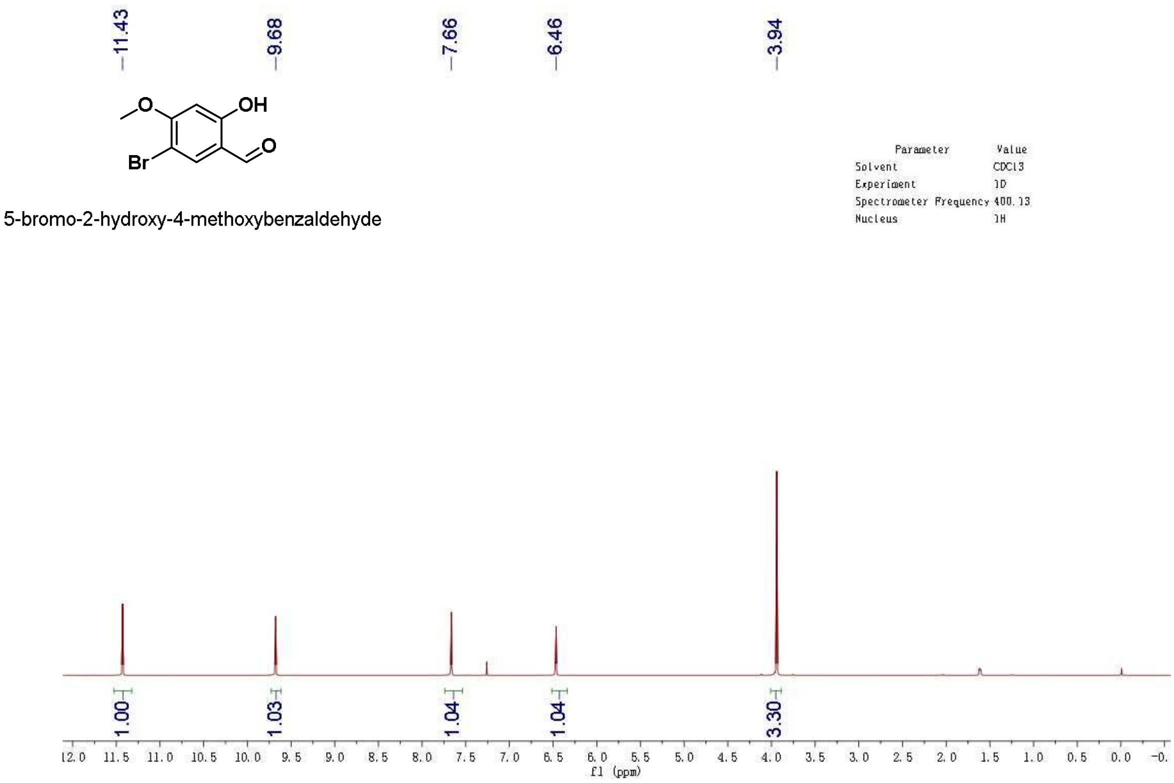

Figure S3. ^{1}H NMR spectrum of 5-bromo-2-hydroxy-4-methoxybenzaldehyde.

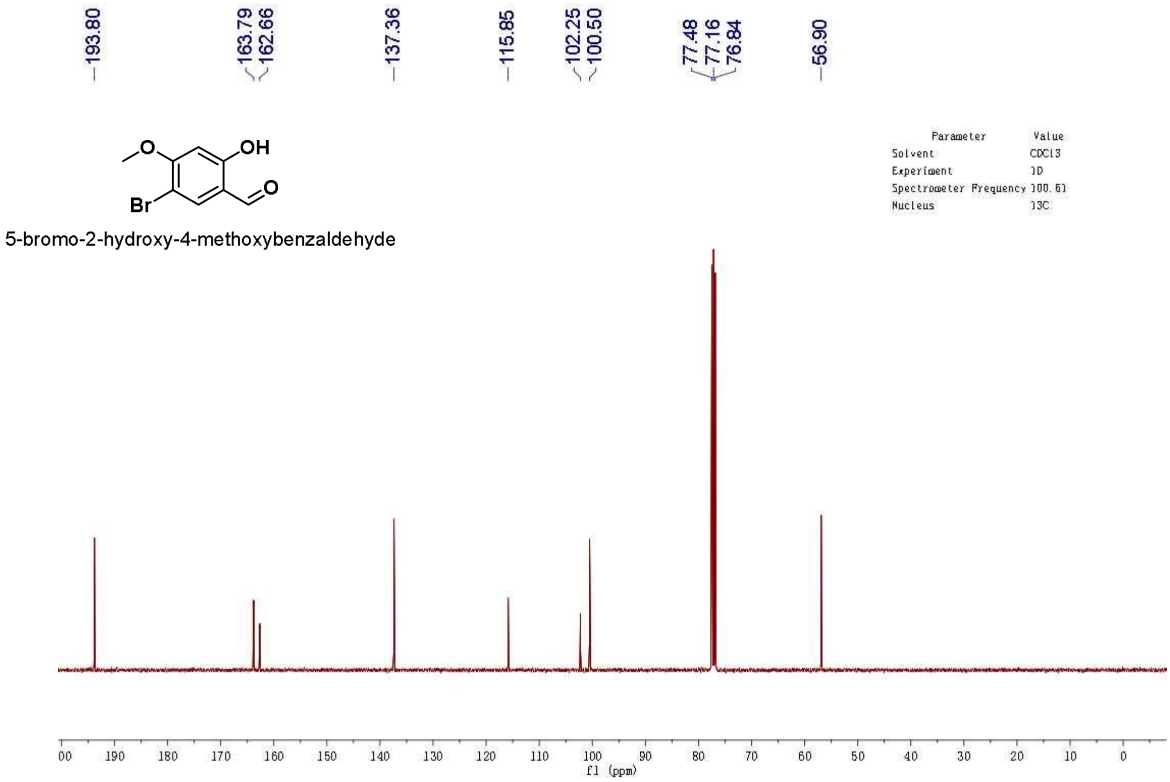

Figure S4. ^{13}C NMR spectrum of 5-bromo-2-hydroxy-4-methoxybenzaldehyde.

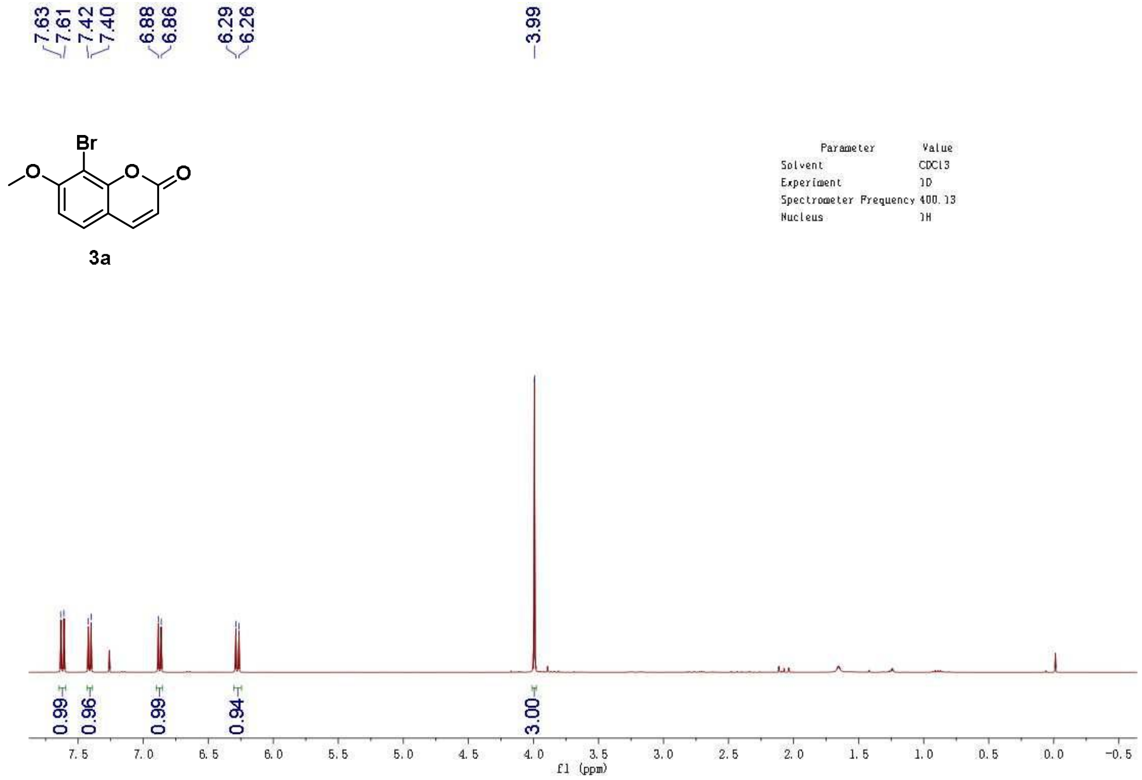

Figure S5. ^{1}H NMR spectrum of compound **3a.**

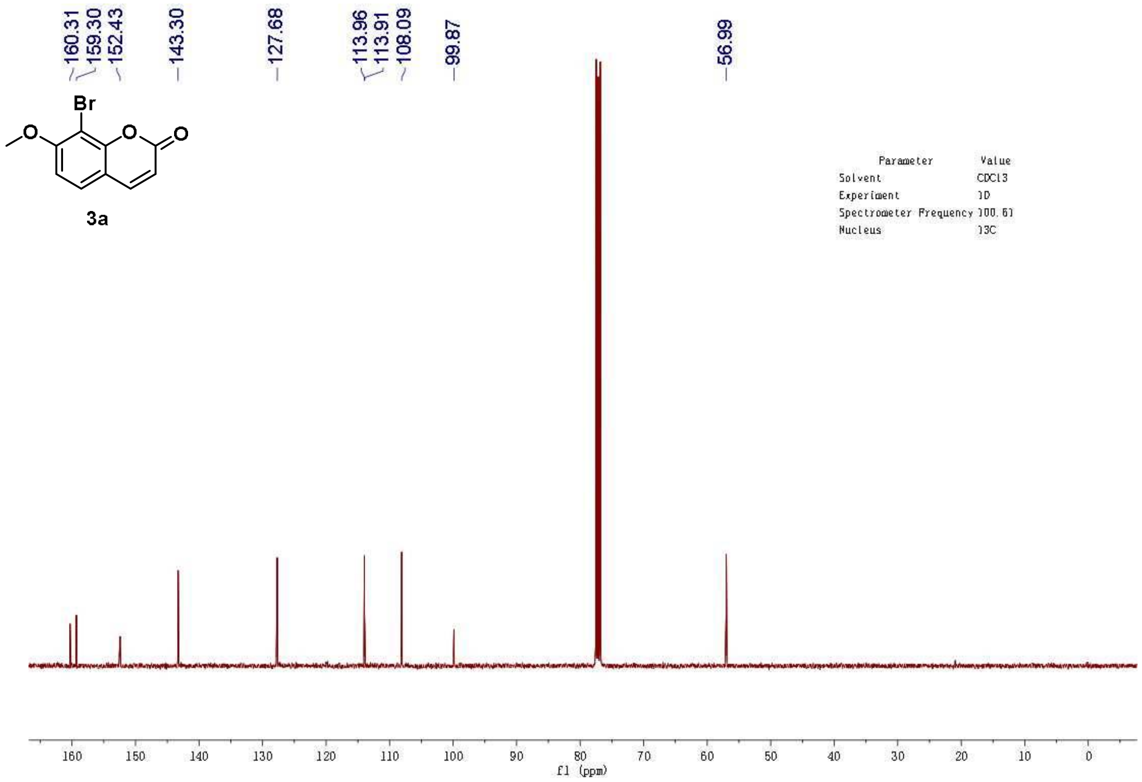

Figure S6. ^{13}C NMR spectrum of compound **3a.**

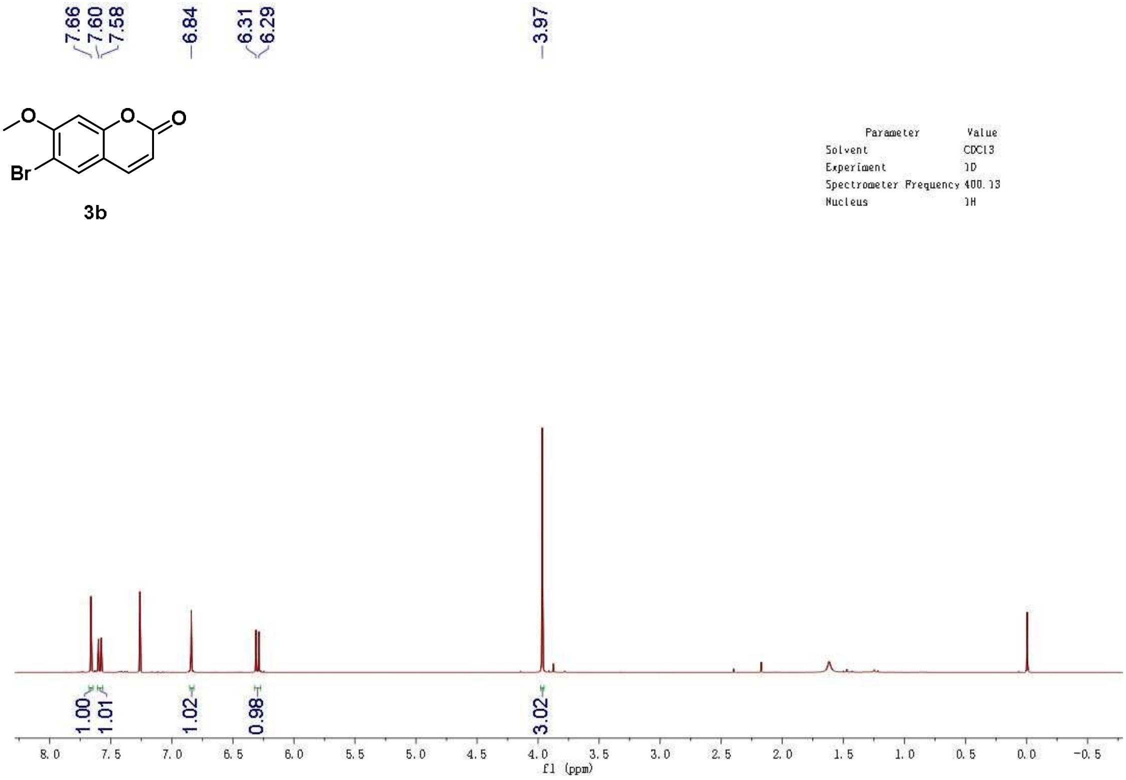

Figure S7. ^{1}H NMR spectrum of compound **3b.**

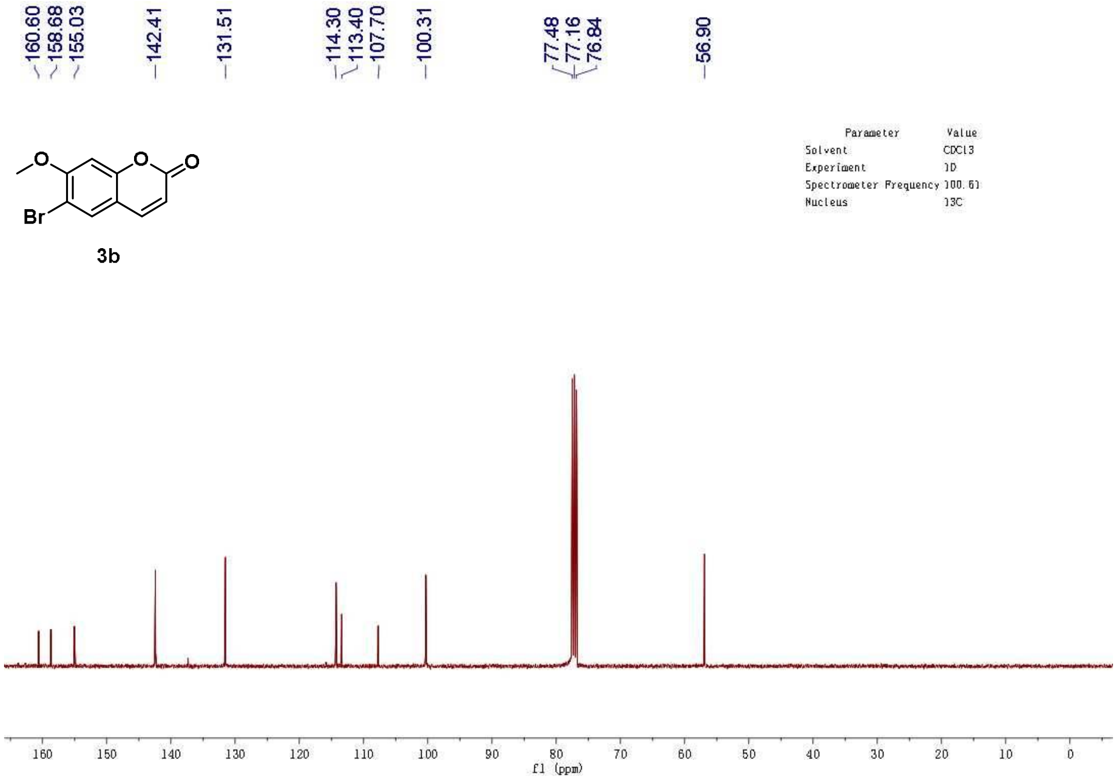

Figure S8. ^{13}C NMR spectrum of compound **3b.**

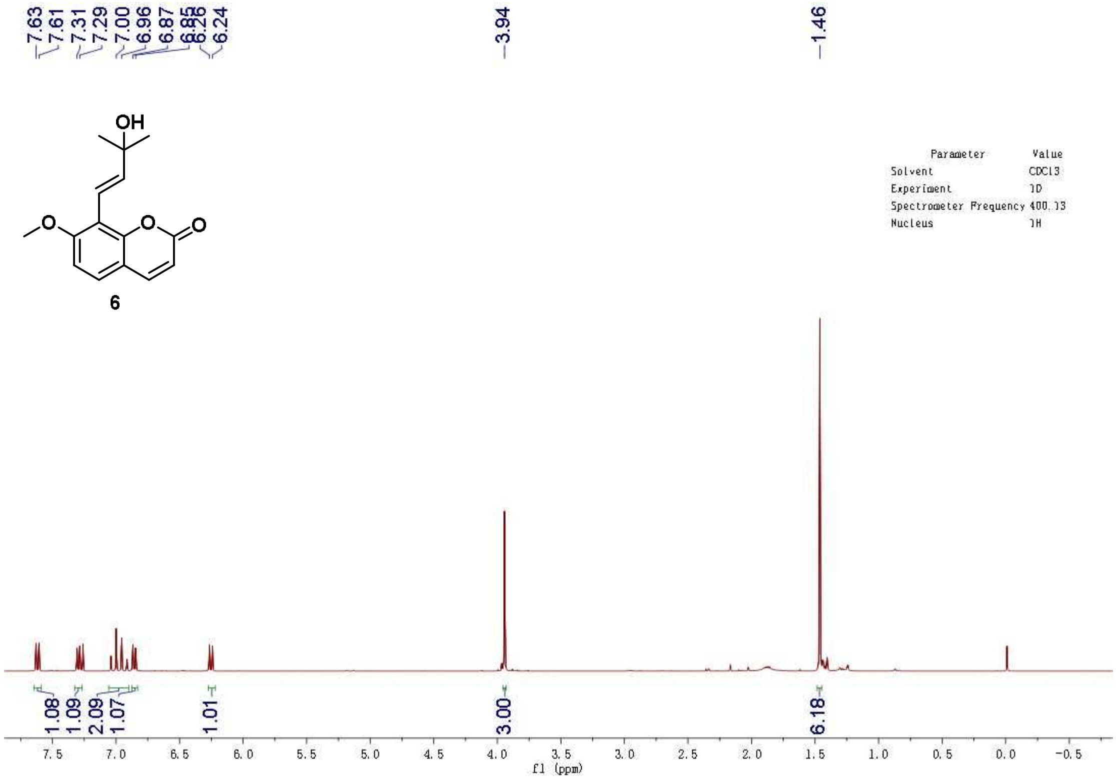

Figure S9. ^{1}H NMR spectrum of compound **6.**

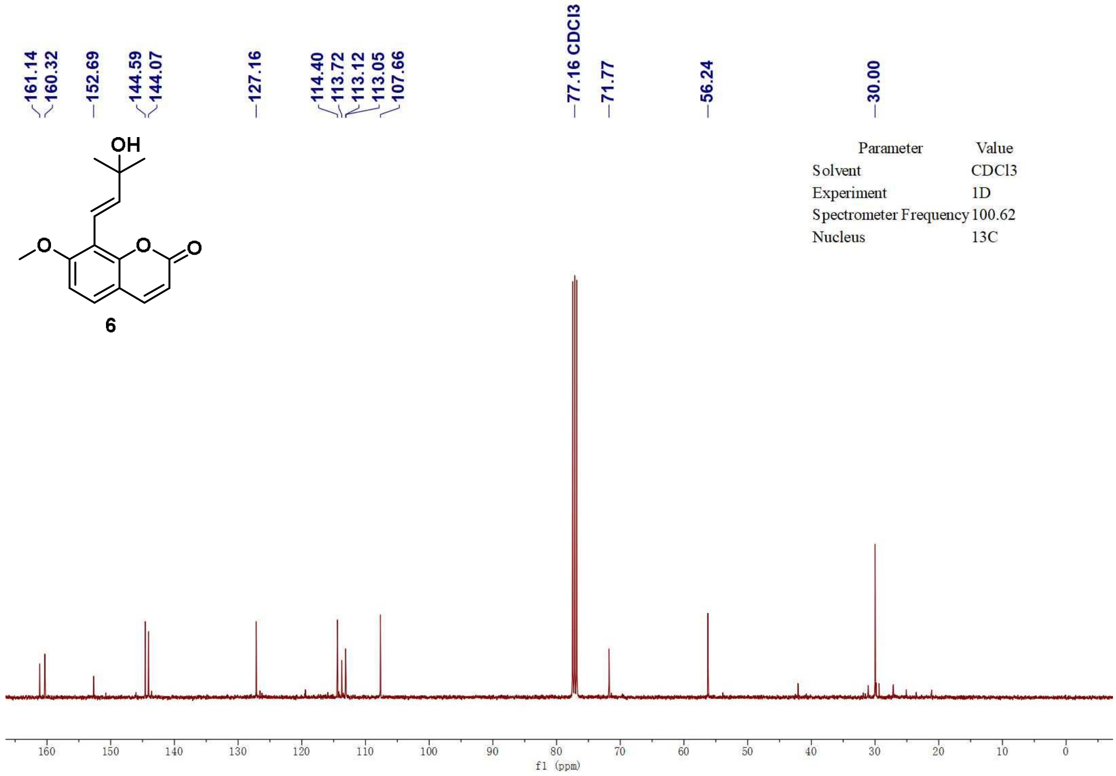

Figure S10. ^{13}C NMR spectrum of compound **6.**

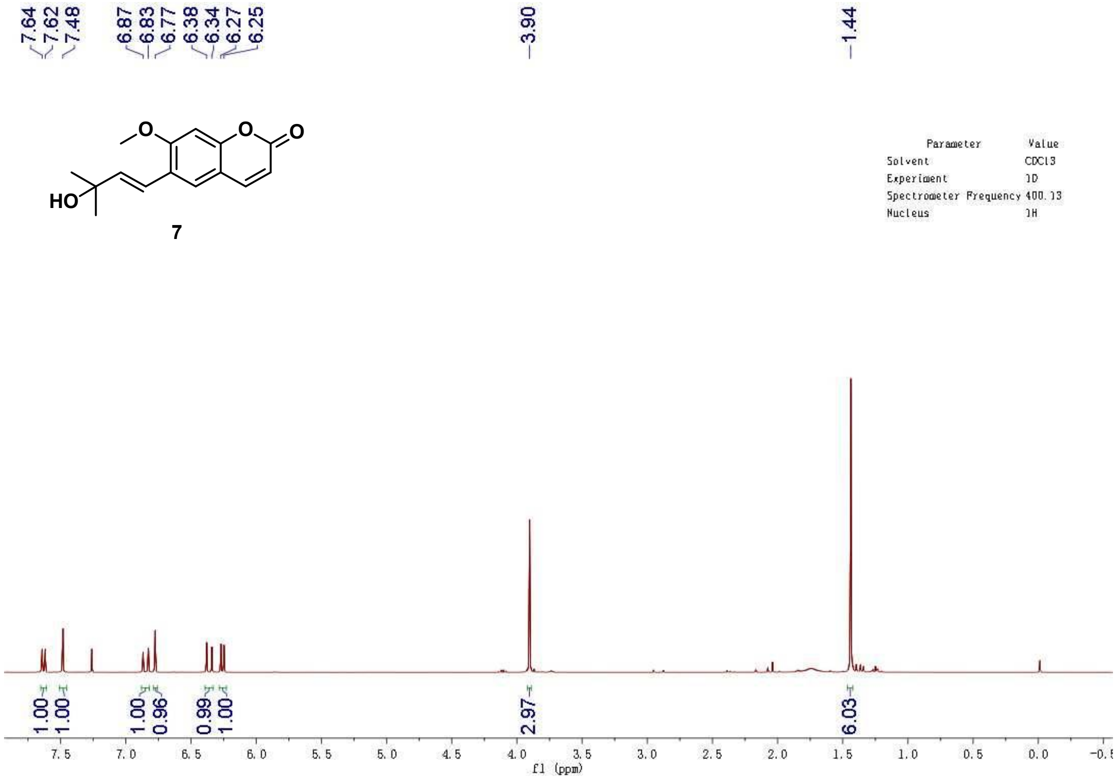

Figure S11. ^{1}H NMR spectrum of compound **7.**

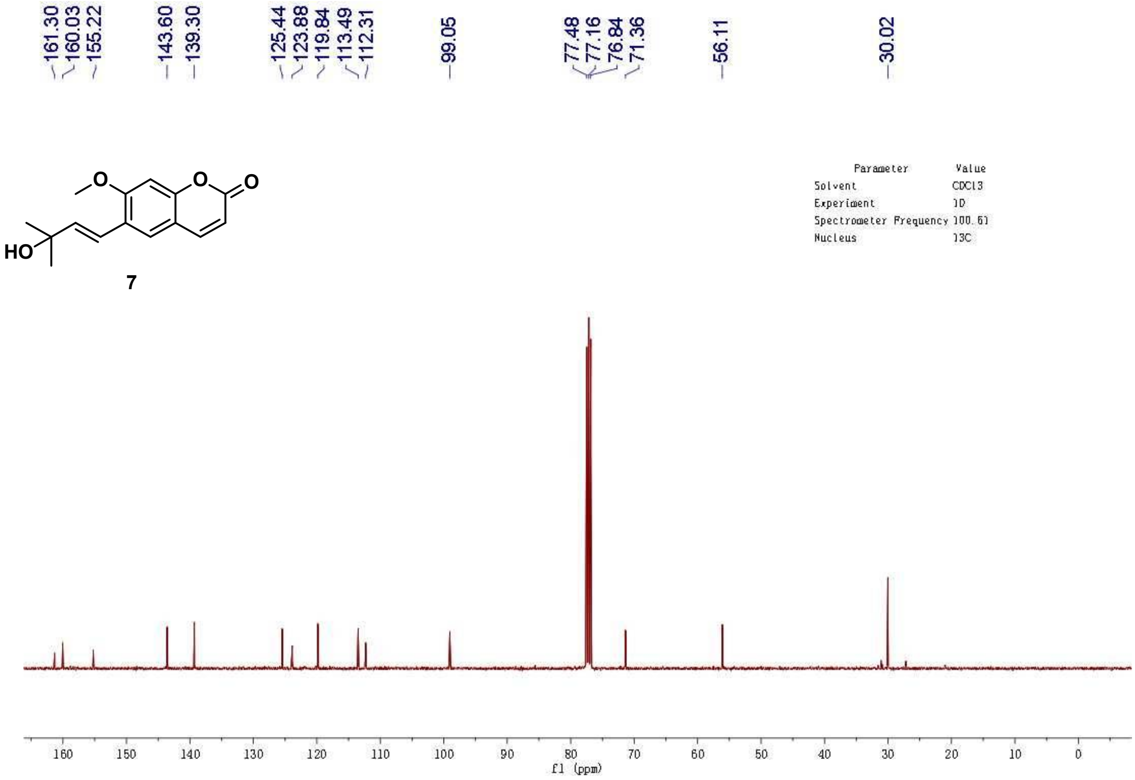

Figure S12. ^{13}C NMR spectrum of compound **7.**

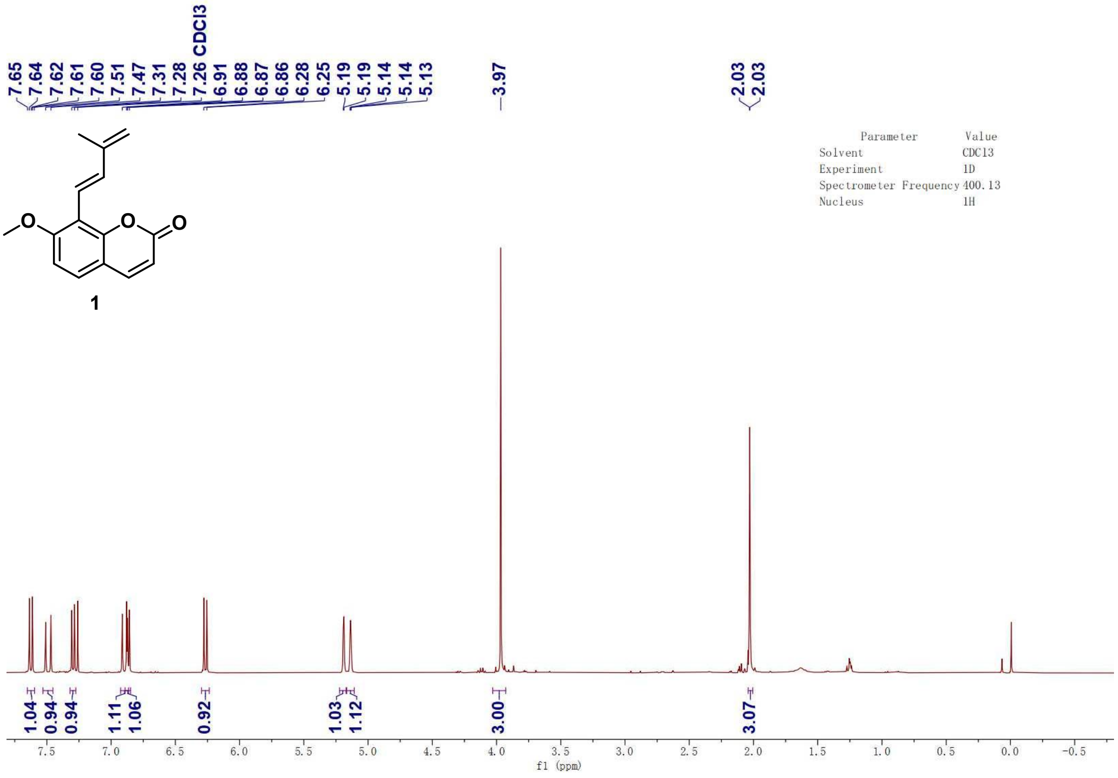

Figure S13. ^{1}H NMR spectrum of *trans*-dehydroosthol **(1).**

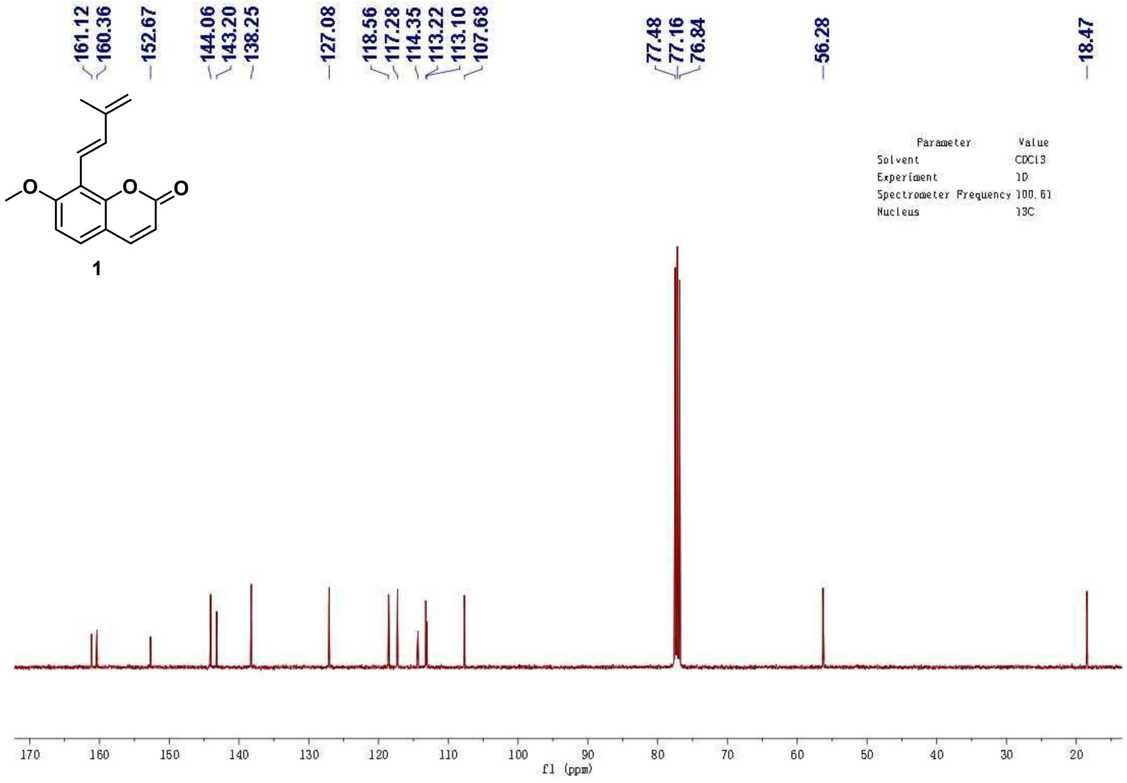

Figure S14. ^{13}C NMR spectrum of *trans*-dehydroosthol **(1).**

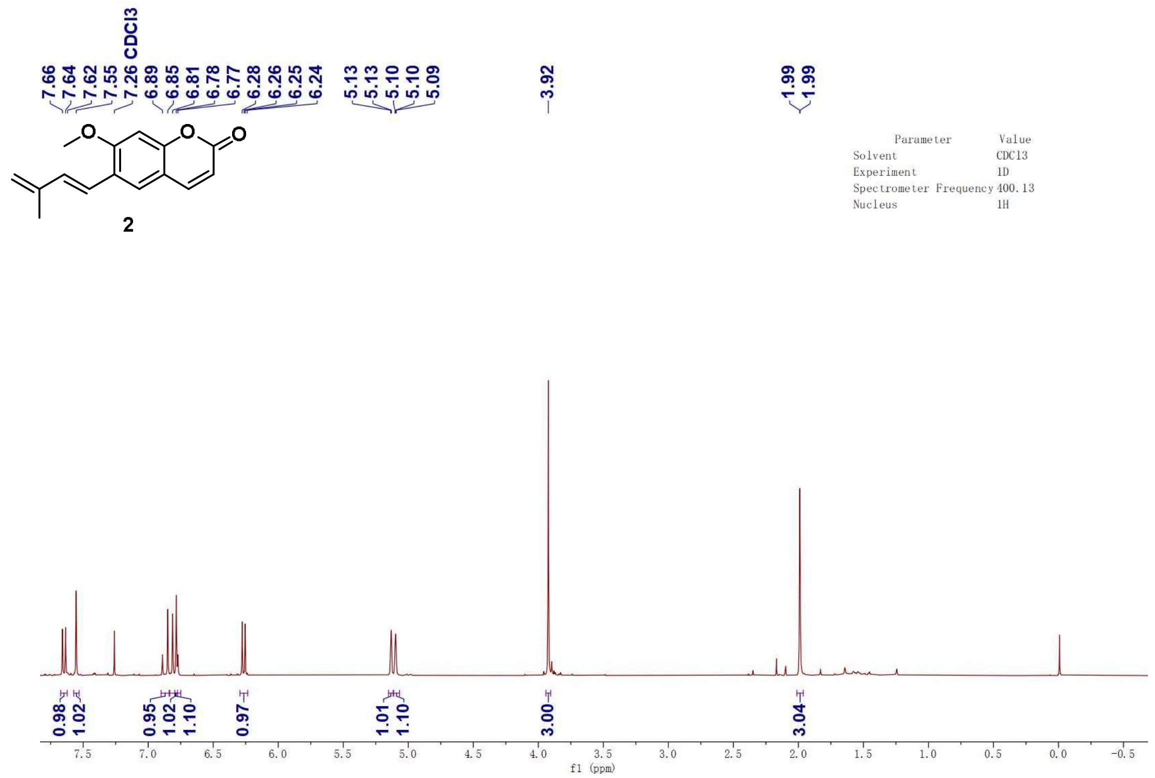

Figure S15. ^{1}H NMR spectrum of citrubuntin **(2).**

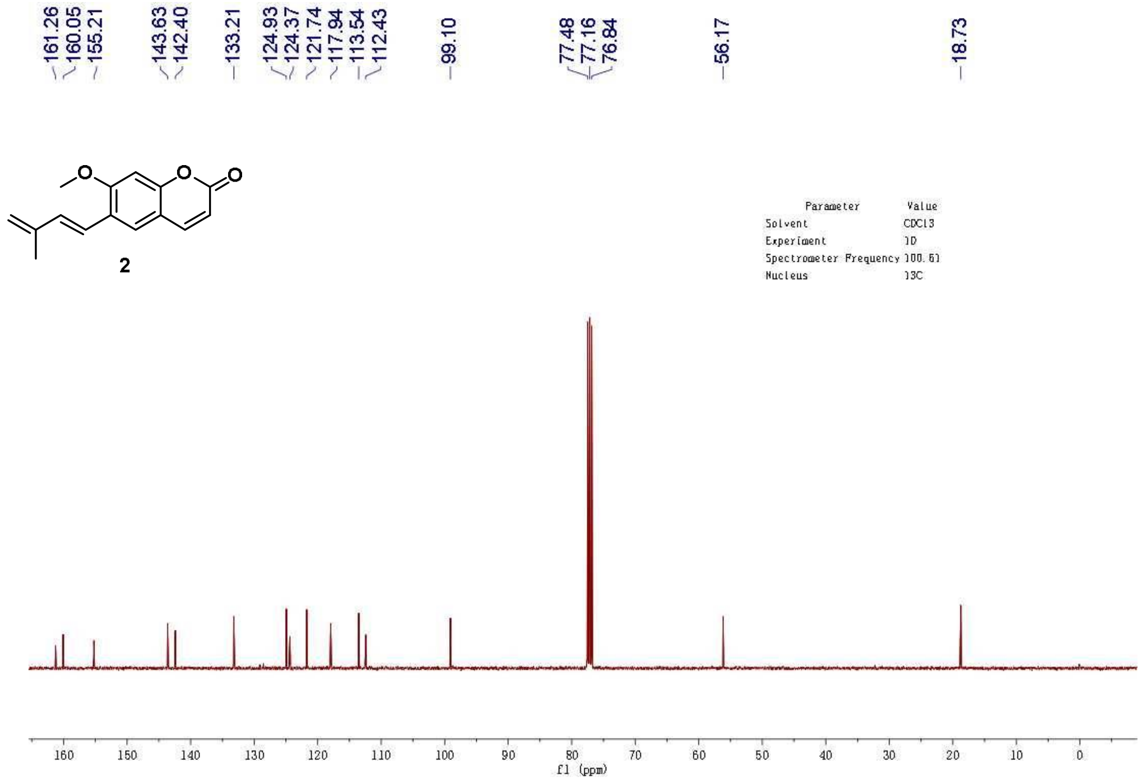

Figure S16. ^{13}C NMR spectrum of citrubuntin **(2).**

Crude ^{1}H NMR spectra of Table 1 (entry 4-1):

trans-dehydroosthol (**1**): ^{1}H NMR (400 MHz, $CDCl_3$) δ_H: 7.62 (d, *J* = 9.6 Hz, 1H), 7.48 (d, *J* = 16.5 Hz, 1H), 7.29 (d, *J* = 8.7 Hz, 1H), 6.94 – 6.83 (m, 2H), 6.26 (d, *J* = 9.4 Hz, 1H), 5.19 (d, *J* = 2.2 Hz, 1H), 5.13 (s, 1H), 3.96 (s, 3H), 2.02 (s, 3H).

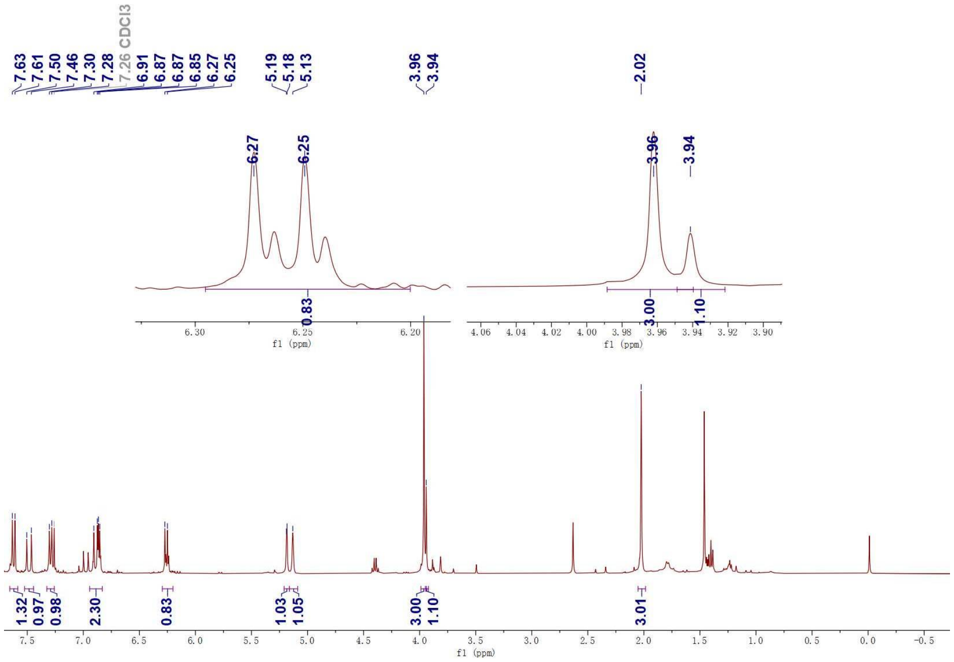

Figure S17. Crude ^{1}H NMR spectra of Table 1 (entry 4-1).

Crude ^{1}H NMR spectra of Table 1 (entry 4-2):

trans-dehydroosthol (**1**): ^{1}H NMR (400 MHz, $CDCl_3$) δ_H: 7.63 (d, *J* = 9.5 Hz, 1H), 7.49 (d, *J* = 16.6 Hz, 1H), 7.30 (d, *J* = 8.6 Hz, 1H), 6.94 – 6.83 (m, 2H), 6.27 (d, *J* = 9.5 Hz, 1H), 5.19 (d, *J* = 2.2 Hz, 1H), 5.14 (t, *J* = 1.8 Hz, 1H), 3.97 (s, 3H), 2.03 (s, 3H).

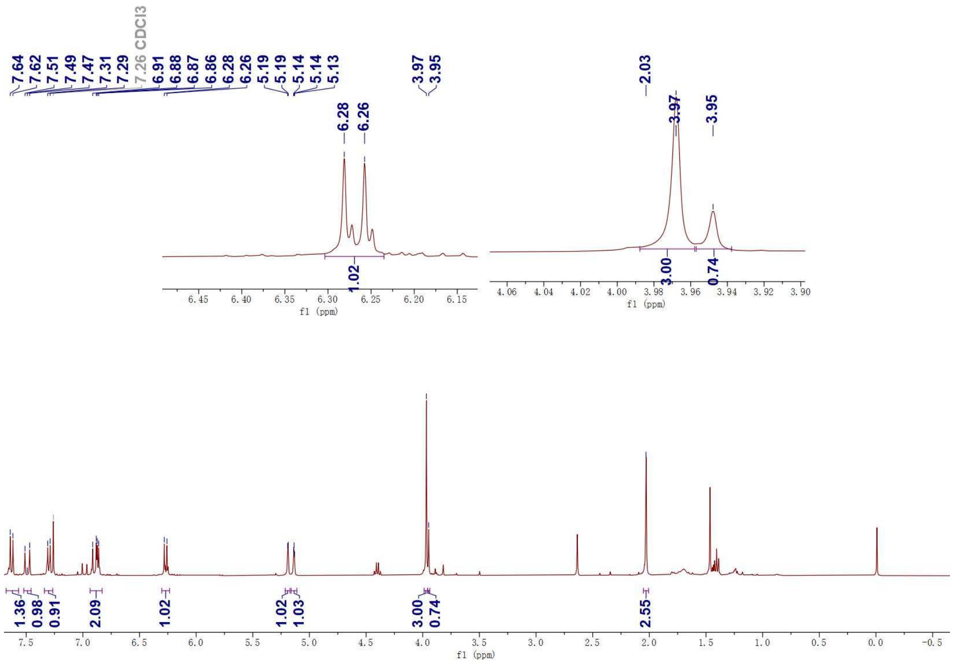

Figure S18. Crude ^{1}H NMR spectra of Table 1 (entry 4-2).

Crude ^{1}H NMR spectra of Table 1 (entry 5-1):

trans-dehydroosthol (**1**): ^{1}H NMR (400 MHz, $CDCl_3$) δ_H: 7.62 (d, *J* = 9.4 Hz, 1H), 7.48 (d, *J* = 16.5 Hz, 1H), 7.29 (d, *J* = 8.7 Hz, 1H), 6.91 – 6.85 (m, 2H), 6.26 (d, *J* = 9.4 Hz, 1H), 5.19 (d, *J* = 2.2 Hz, 1H), 5.13 (t, *J* = 1.9 Hz, 1H), 3.96 (s, 3H), 2.02 (d, *J* = 1.2 Hz, 3H).

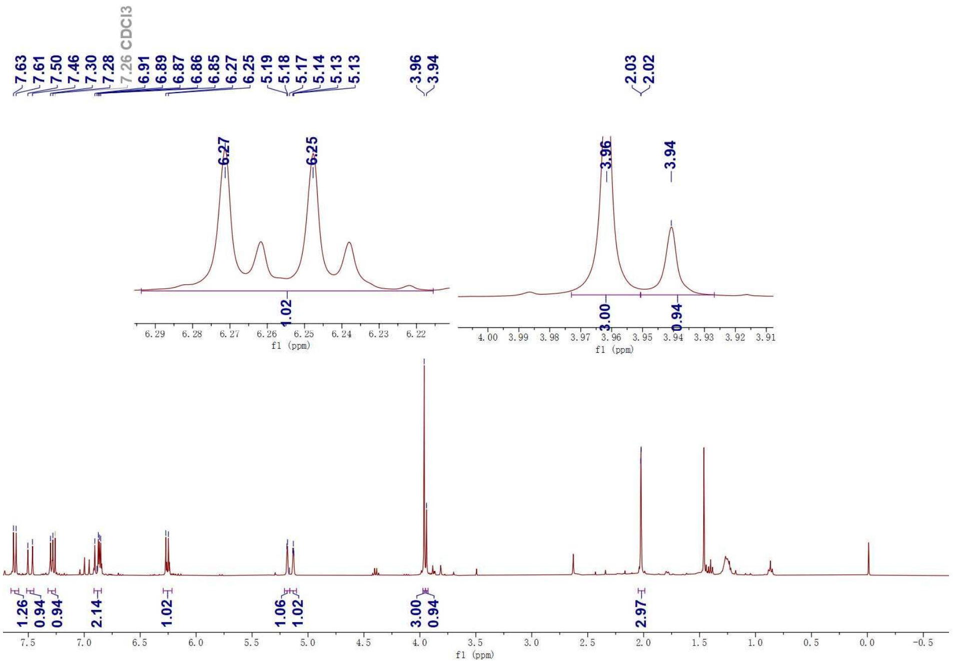

Figure S19. Crude ^{1}H NMR spectra of Table 1 (entry 5-1).

Crude ^{1}H NMR spectra of Table 1 (entry 5-2):

trans-dehydroosthol (**1**): ^{1}H NMR (400 MHz, $CDCl_3$) δ_H: 7.62 (d, *J* = 9.5 Hz, 1H), 7.48 (d, *J* = 16.5 Hz, 1H), 7.29 (d, *J* = 8.6 Hz, 1H), 6.93 – 6.83 (m, 2H), 6.26 (d, *J* = 9.5 Hz, 1H), 5.19 (d, *J* = 2.2 Hz, 1H), 5.13 (s, 1H), 3.96 (s, 3H), 2.02 (s, 3H).

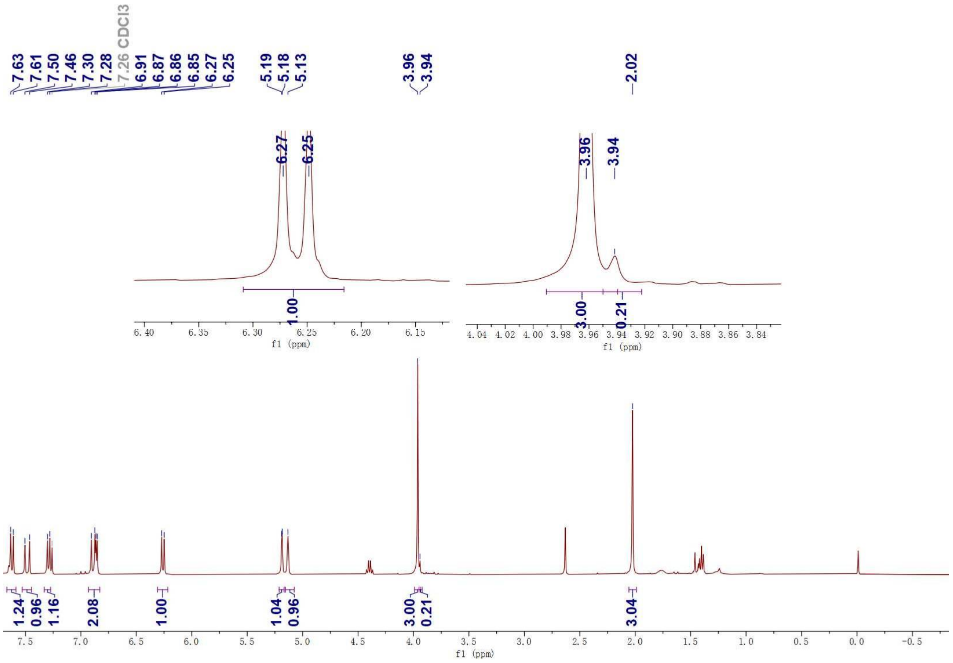

Figure S20. Crude ^{1}H NMR spectra of Table 1 (entry 5-2).

Crude ^{1}H NMR spectra of Table 2 (entry 6-1):

trans-dehydroosthol (**1**): ^{1}H NMR (400 MHz, $CDCl_3$) δ_H: 7.62 (d, *J* = 9.5 Hz, 1H), 7.48 (d, *J* = 16.5 Hz, 1H), 7.29 (d, *J* = 8.6 Hz, 1H), 6.93 – 6.83 (m, 2H), 6.26 (d, *J* = 9.4 Hz, 1H), 5.19 (d, *J* = 2.1 Hz, 1H), 5.13 (d, *J* = 2.1 Hz, 1H), 3.96 (s, 3H), 2.03 (s, 3H).

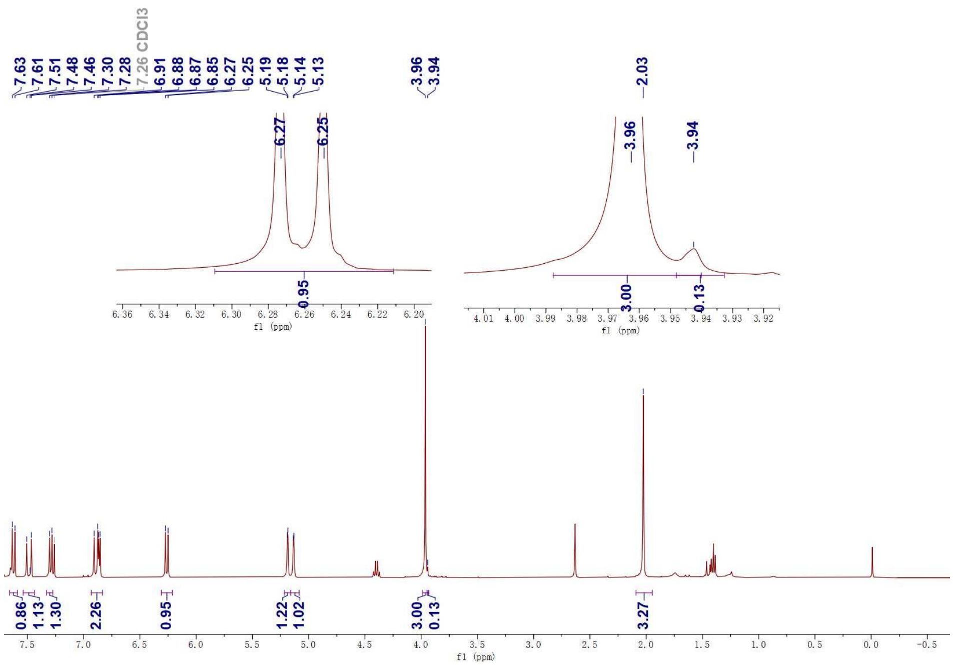

Figure S21. Crude ^{1}H NMR spectra of Table 2 (entry 6-1).

Crude ^{1}H NMR spectra of Table 2 (entry 6-2):

trans-dehydroosthol (**1**): ^{1}H NMR (400 MHz, $CDCl_3$) δ_H: 7.62 (d, *J* = 9.5 Hz, 1H), 7.49 (d, *J* = 16.5 Hz, 1H), 7.29 (d, *J* = 8.6 Hz, 1H), 6.92 – 6.84 (m, 2H), 6.26 (d, *J* = 9.5 Hz, 1H), 5.19 (d, *J* = 2.3 Hz, 1H), 5.13 (t, *J* = 1.9 Hz, 1H), 3.96 (s, 3H), 2.04 (s, 3H).

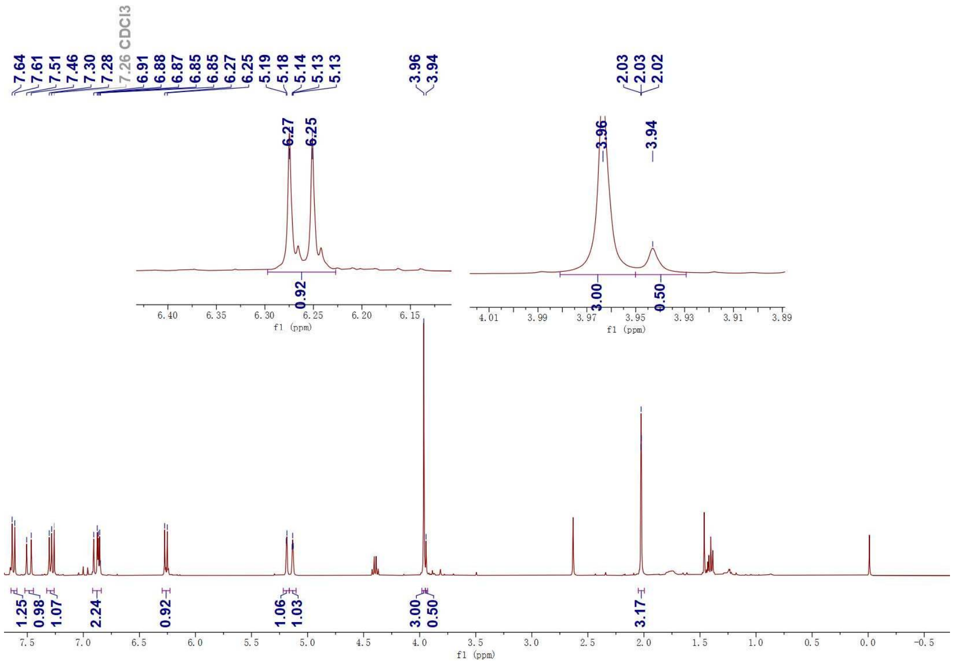

Figure S22. Crude ^{1}H NMR spectra of Table 2 (entry 6-2).

Crude ^{1}H NMR spectra of Table 3 (entry 2-1):

trans-dehydroosthol (**1**): ^{1}H NMR (400 MHz, $CDCl_3$) δ_H: 7.62 (d, *J* = 9.4 Hz, 1H), 7.49 (d, *J* = 16.5 Hz, 1H), 7.29 (d, *J* = 8.7 Hz, 1H), 6.94 – 6.82 (m, 2H), 6.26 (d, *J* = 9.5 Hz, 1H), 5.19 (d, *J* = 2.2 Hz, 1H), 5.13 (s, 1H), 3.96 (s, 3H), 3.94 (s, 0H), 2.03 (s, 3H).

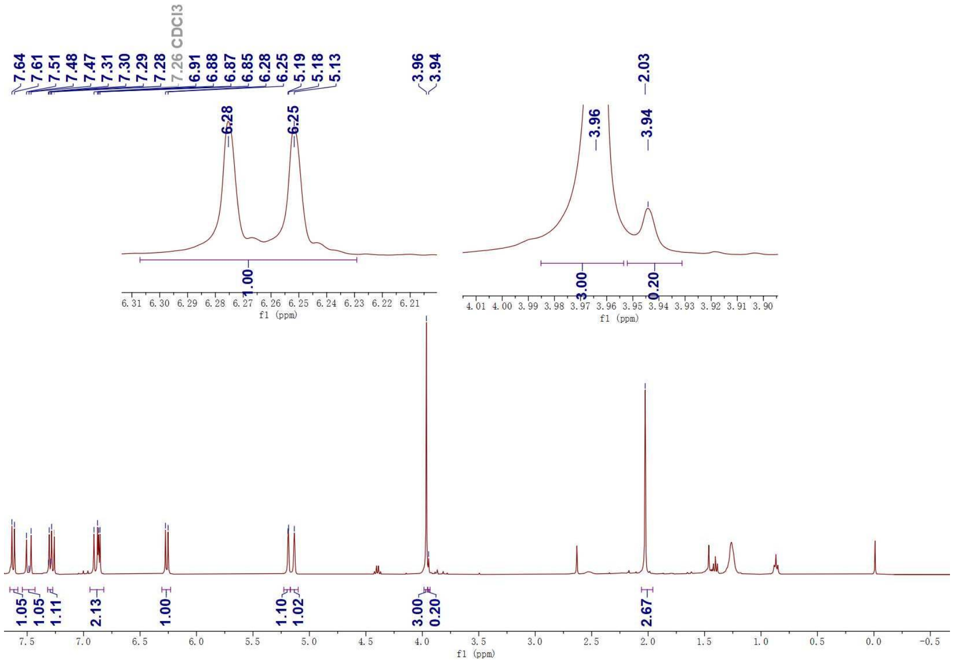

Figure S23. Crude ^{1}H NMR spectra of Table 3 (entry 2-1).

Crude ^{1}H NMR spectra of Table 3 (entry 2-2):

trans-dehydroosthol (**1**): ^{1}H NMR (400 MHz, $CDCl_3$) δ_H: 7.61 (d, *J* = 9.5 Hz, 1H), 7.47 (d, *J* = 16.5 Hz, 1H), 7.28 (d, *J* = 8.7 Hz, 1H), 6.92 – 6.80 (m, 2H), 6.25 (d, *J* = 9.5 Hz, 1H), 5.18 (d, *J* = 2.2 Hz, 1H), 5.13 (t, *J* = 1.8 Hz, 1H), 3.95 (s, 3H), 2.02 (s, 3H).

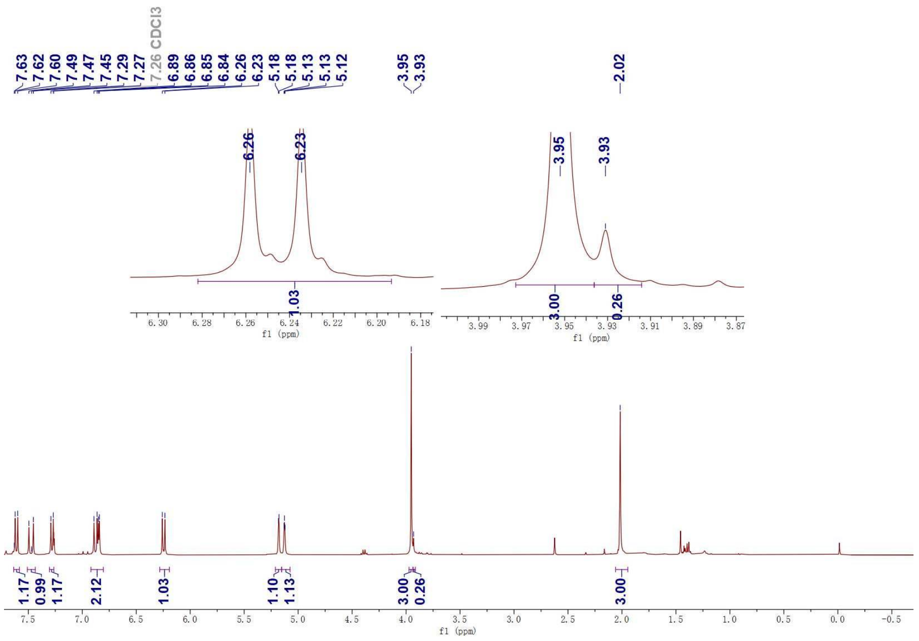

Figure S24. Crude ^{1}H NMR spectra of Table 3 (entry 2-2).

^{1}H and ^{13}C NMR spectral comparisons for compounds

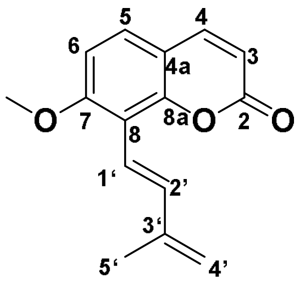

1

Table S1. ^{1}H and ^{13}C NMR data of synthetic **1** and literature **1.**

Position	Synthetic 1[a]		Literature 1[b]	
	δ_H (ppm)	δ_C (ppm)	δ_H (ppm)	δ_C (ppm)
1				
2		161.12		-
3	6.27 (d, J = 9.5 Hz, 1H)	113.22	6.27 (d, J = 9.4 Hz, 1H)	-
4	7.62 (d, J = 9.5 Hz, 1H)	144.06	7.63 (d, J = 9.4 Hz, 1H)	-
5	7.30 (d, J = 8.6 Hz, 1H)	127.08	7.30 (d, J = 8.7 Hz, 1H)	-
6	6.86 (d, J = 8.6 Hz, 1H)	107.68	6.87 (d, J = 8.7 Hz, 1H)	-
7		160.36		-
8		118.56		-

8a		152.67		-
4a		113.10		-
1’	7.49 (d, *J* = 16.5 Hz, 1H)	114.35	7.49 (d, *J* = 16.8 Hz, 1H)	-
2’	6.90 (d, *J* = 16.5 Hz, 1H)	138.25	6.90 (d, *J* = 16.8 Hz, 1H)	-
3’		143.20		-
4’	5.19 (d, *J* = 2.2 Hz, 1H) 5.14 (t, *J* = 1.8 Hz, 1H)	117.28	5.19 (s, 1H) 5.14 (s, 1H)	-
5’	2.03 (d, *J* = 1.3 Hz, 3H)	18.47	2.03 (s, 3H, CH_3)	-
-OCH_3	3.97 (s, 3H)	56.28	3.97 (s, 3H)	-

[a] ^{1}H (400 MHz) and ^{13}C NMR (100 MHz) in $CDCl_3$.

[b] ^{1}H (400 MHz) and ^{13}C NMR (100 MHz) in $CDCl_3$ are taken from ref 9.

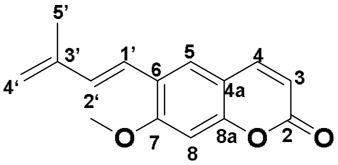

2

Table S2. ^{1}H and ^{13}C NMR data of synthetic **2** and literature **2.**

Position	Synthetic **2**[a]		Literature **2**[b]	
	δ_H (ppm)	δ_C (ppm)	δ_H (ppm)	δ_C (ppm)
1				
2		161.26		161.31
3	6.27 (d, *J* = 9.5 Hz, 1H)	113.54	6.24 (d, *J* = 9.5 Hz,1H)	113.46
4	7.65 (d, *J* = 9.5 Hz, 1H)	142.40	7.63 (d, *J* = 9.5 Hz, 1H)	143.75
5	7.55 (s, 1H)	124.93	7.51(s, lH)	125.01
6		124.37		124.36
7		160.05		160.21
8	6.78 (s, 1H)	99.10	6.72 (s, 1 H)	99.02
8a		155.21		155.33
4a		112.43		112.45
1’	6.79 (d, *J* =16.3 Hz, 1H)	121.74	6.76 (d, *J* =16.2 Hz, 1H)	121.8
2’	6.87 (d, *J* =16.3 Hz, 1H)	133.21	6.87 (d, *J* = 16.2 Hz, 1H)	133.23

3’		143.63		142.51
4’	5.13 (d, *J* = 2.1 Hz, 1H)<br>5.10 (t, *J* = 1.7 Hz, 1H)	117.94	5.12 (m, 2H)	117.93
5’	1.99 (d, *J* = 1.2 Hz, 3H)	18.73	2.00(s, 3H, CH_3)	18.45
-OCH_3	3.92 (s, 3H)	56.17	3.90(s, 3H)	56.01

[a] ^{1}H (400 MHz) and ^{13}C NMR (100 MHz) in $CDCl_3$.

[b] ^{1}H (400 MHz) and ^{13}C NMR (100 MHz) in $CDCl_3$ are taken from ref 17.

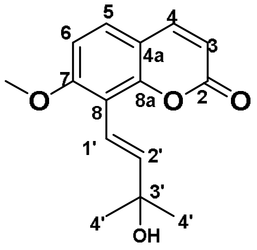

6

Table S3. ^{1}H and ^{13}C NMR data of synthetic **6** and literature **6.**

Position	Synthetic **6**[a]		Literature **6**[b]	
	δ_H (ppm)	δ_C (ppm)	δ_H (ppm)	δ_C (ppm)
1				
2		161.14		161.0
3	6.25 (d, *J* = 9.4 Hz, 1H)	113.12	6.25 (d, *J* = 9.4 Hz, 1H)	113.3
4	7.62 (d, *J* = 9.4 Hz, 1H)	144.59	7.62 (d, *J* = 9.5 Hz, 1H)	144.6
5	7.30 (d, *J* = 8.6 Hz, 1H)	127.16	7.30 (d, *J* = 8.6 Hz, 1H)	127.2
6	6.86 (d, *J* = 8.6 Hz, 1H)	107.66	6.86 (d, *J* = 8.7 Hz, 1H)	107.7
7		160.32		160.4
8		113.05		113.1
8a		152.69		152.8
4a		113.72		113.8
1’	7.02 (d, 1H, *J* = 16.5 Hz)	114.40	7.02 (d, *J* = 16.5 Hz, 1H)	114.5
2’	6.93 (d, 1H, *J* = 16.5 Hz)	144.07	6.94 (d, *J* = 16.5 Hz, 1H)	144.0
3’		71.77		71.9
4’	1.46 (s, 6H)	30.00	1.47 (s, 6H, 2(CH_3))	30.1

-OCH_3	3.94 (s, 3H)	56.24	3.95 (s, 3H)	56.3

[a] ^1H (400 MHz) and ^{13}C NMR (100 MHz) in $CDCl_3$.

[b] ^1H (400 MHz) and ^{13}C NMR (100 MHz) in $CDCl_3$ are taken from ref 21 and ref 27.

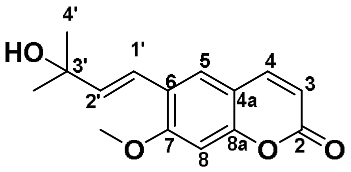

7

Table S4. ^1H and ^{13}C NMR data of synthetic **7** and literature **7.**

Position	Synthetic **7**[a]		Literature **7**[b]	
	δ_H (ppm)	δ_C (ppm)	δ_H (ppm)	δ_C (ppm)
1				
2		161.30		161.2
3	6.26 (d, J = 9.5 Hz, 1H)	113.49	6.26 (d, J = 9.4 Hz, 1H)	113.6
4	7.63 (d, J = 9.5 Hz, 1H)	143.60	7.63 (d, J = 9.5 Hz, 1H)	143.6
5	7.48 (s, 1H)	119.84	7.49 (s, 1H)	119.9
6		123.88		123.9
7		160.03		160.1
8	6.77 (s, 1H)	99.05	6.79 (s, 1H)	99.1
8a		155.22		155.3
4a		112.31		112.4
1’	6.85 (d, J = 16.2 Hz, 1H)	125.44	6.86 (d, J = 16.2 Hz, 1H)	125.5
2’	6.36 (d, J = 16.2 Hz, 1H)	139.30	6.37 (d, J = 16.2 Hz, 1H)	139.3
3’		71.36		71.4
4’	1.44 (s, 6H)	30.02	1.44 (s, 6H, 2(CH_3))	30.1
-OCH_3	3.90 (s, 3H)	56.11	3.91 (s, 3H)	56.1

[a] ^1H (400 MHz) and ^{13}C NMR (100 MHz) in $CDCl_3$.

[b] ^1H (400 MHz) and ^{13}C NMR (100 MHz) in $CDCl_3$ are taken from ref. 27.

Reference

[9] Ito, C.; Furukawa, H. Constituents of *Murraya exotica L.* structure elucidation of new coumarins. *Chem. Pharm. Bull.* **1987**, *35*, 4277–4285.

[17] Reisch, J.; Bathe, A. Naturstoffchemie, 118[1)] synthese der cumarine 6- und 8-naphthoherniarin, dehydrogeijerin und Murraol. *Liebigs Ann. Chem.* **1988**, *1988*, 543–547.

[21] Reisch, J.; Herath, H.M.T.B.; Kumar, N.S. ChemInform abstract: Natural product chemistry. Part 139. Synthesis of the natural coumarins (E)-suberenol, cyclobisuberodiene and two other related new coumarins. *Liebigs. Ann. Chem.* **1990**, *1990*, 931–933.

[27] Guthertz, A.; Leutzsch, M.; Wolf, L.M.; Gupta, P.; Rummelt, S.M.; Goddard, R.; Farès, C.; Thiel, W.; Fürstner, A. Half-sandwich ruthenium carbene complexes link trans-hydrogenation and gem-hydrogenation of internal alkynes. *J. Am. Chem. Soc.* **2018**, *140*, 3156–3169.
